# Cell‐Type Specific miRNA Regulatory Network Responses to ABA Stress Revealed by Time Series Transcriptional Atlases in *Arabidopsis*

**DOI:** 10.1002/advs.202415083

**Published:** 2025-01-10

**Authors:** Zhaoxu Gao, Yanning Su, Guanzhong Jiao, Zhiying Lou, Le Chang, Renbo Yu, Chao Xu, Xue Han, Zejia Wang, Jian Li, Xing Wang Deng, Hang He

**Affiliations:** ^1^ School of Advanced Agriculture Sciences and School of Life Sciences State Key Laboratory of Protein and Plant Gene Research Peking University Beijing 100871 China; ^2^ Institute of Crop Science Chinese Academy of Agricultural Sciences (CAAS) Beijing 100081 China; ^3^ Peking University Institute of Advanced Agricultural Sciences Shandong Laboratory of Advanced Agricultural Sciences in Weifang Shandong 261325 China; ^4^ Tropical Crops Genetic Resources Institute Chinese Academy of Tropical Agricultural Sciences Haikou 571101 China

**Keywords:** cell‐type specific, crosstalk, dynamic, M‐FFLs, miRNAs, network, rapid, scRNA‐seq

## Abstract

In plants, microRNAs (miRNAs) participate in complex gene regulatory networks together with the transcription factors (TFs) in response to biotic and abiotic stresses. To date, analyses of miRNAs‐induced transcriptome remodeling are at the whole plant or tissue levels. Here, *Arabidopsis*’s ABA‐induced single‐cell RNA‐seq (scRNA‐seq) is performed at different stages of time points–early, middle, and late. Single‐cell level primary miRNAs (pri‐miRNAs) atlas supported the rapid, dynamic, and cell‐type specific miRNA responses under ABA treatment. MiRNAs respond rapidly and prior to target gene expression dynamics, and these rapid response miRNAs are highly cell‐type specific, especially in mesophyll and vascular cells. MiRNA‐TF‐mRNA regulation modules are identified by identifying miRNA‐contained feed‐forward loops (M‐FFLs) in the regulatory network, and regulatory networks with M‐FFLs have higher co‐expression and clustering coefficient (CC) values than those without M‐FFLs, suggesting the hub role of miRNAs in regulatory networks. The cell‐type‐specific M‐FFLs are regulated by these hub miRNAs rather than TFs through sc‐RNA‐seq network analysis. MiR858a‐FBH3‐MYB module inhibited the expression of MYB63 and MYB20, which related to the formation of plant secondary wall and the production of lignin, through M‐FFL specifically in vascular. These results can provide prominent insights into miRNAs' dynamic and cell‐type‐specific roles in plant development and stress responses.

## Graphical abstract

This integrated network study offers insights into miRNA regulation, impacting plant development and cell differentiation. MiRNAs and pri‐miRNAs show dynamic, cell‐type‐specific ABA responses, especially in the early time‐point. The TF‐miRNA‐target network (TMTN) reveals that miRNAs enhance co‐expression with cell‐type‐specific roles. MiR858a inhibits MYB63 and MYB20 in vascular cells by MiR858a‐FBH3‐MYB loop in TMTN, affecting the secondary wall and lignin formation.

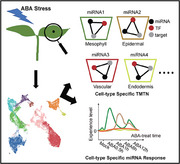

## 1 Introduction

MiRNAs are small non‐coding RNAs that play an important role in the post‐transcriptional regulation of organisms. The main regulatory role of miRNA is to inhibit the expression of the genes through the degradation of transcripts or to inhibit the process of translation.^[^ ^1^ ^]^ Parallel analysis of RNA ends (PARE) can find the miRNA targets with transcript cleavage.^[^ ^2^ ^]^ Some functions of translational inhibition exist for the disproportionate amount of miRNAs and transcripts of their target genes.^[^ ^3^, ^4^, ^5^ ^]^ In addition to its traditional role, miRNA possesses the following unconventional functions: the mobility and tissue specificity of miRNAs. In plants, it was found that miRNAs can produce unidirectional movement from bud to root in interspecific heterograft.^[^ ^6^ ^]^ The ectomycorrhizal fungus *Pisolithus microcarpus* can transfer miRNA into host cells and regulate host gene expression to promote symbiosis.^[^ ^7^ ^]^ Also, miRNAs can be transferred from parasitic plants to their hosts (cross species), where they can disrupt processes such as the host's self‐defense ability.^[^ ^8^ ^]^ Besides, miRNAs also have some rare functions that play a role in specific situations: encoding peptides (primary (pri)‐miRNA); targeting mitochondrial transcripts; directly activating transcription and so on.^[^ ^9^ ^]^

MiRNA plays a crucial role in regulating the expression of genes involved in many developmental processes and in responding to external factors. Some miRNAs play multiple roles in plants, and some processes are regulated by multiple miRNAs. In previous studies, *miR156* plays important roles in the development and response of abiotic stress.^[^ ^10^ ^]^ Moreover, *miR160*, *miR167*, *miR165*, *miR166*, and *miR169* may also play a role in the *Arabidopsis* drought response.^[^ ^11^, ^12^, ^13^, ^14^, ^15^, ^16^, ^17^ ^]^ However, most of the miRNA‐target modules in plant‐environment interactions are verified solely by experiment. In our previous analysis, the target genes in miRNA modules were shown to be enriched in responses to abiotic/biotic stimuli, development, and gene expression.^[^ ^18^ ^]^ Similar to other genes, RNA polymerase II (Pol II) regulates the transcription of miRNA genes into pri‐miRNA.^[^ ^19^, ^20^ ^]^ At the same time, miRNA genes are regulated by other factors; thus, miRNA is an important element in biological networks.

When plants face stress, different genes and regulatory factors collaborate through a network. Network walking of nitrogen signaling has defined a dynamic transcriptional network by integrating validated and predicted TF‐target interactions.^[^ ^21^ ^]^ One classical example is a hierarchical ABA response network. It was constructed using time series RNA‐seq and ChIP‐seq data of 21 TFs, which revealed the systematic regulation of abiotic responses.^[^ ^22^ ^]^ TFs and miRNAs are two primary classes of regulators within the network that possess defined target specificity.^[^ ^23^ ^]^ *SEPALLATA3‐miR319‐TCP* feed‐forward loop (FFL) has been found in the gene regulatory network (GRN) that controls the early stages of flower development. The GRN constructed using binding and expression data for TFs and miRNAs has demonstrated that a combination of TFs and miRNAs is important for biological process analysis.^[^ ^24^ ^]^

The organism itself is dynamic, so the analysis of a static network can't satisfy the real regulation law of the organism. As the genetic regulation of gene expression is dynamic, the expression of quantitative trait loci (eQTLs) is also dynamic in different cell types. Some specific eQTLs are associated with specialized function.^[^ ^25^ ^]^ Many existing reports show that the time series RNA‐seq data could identify key regulators of different cell types or dynamic development process.^[^ ^26^, ^27^, ^28^ ^]^ The dynamic miRNAs and miRNA‐directed repression of multiple transcription factors are required for embryo morphogenesis.^[^ ^29^ ^]^ Not only the report of time series miRNAs but also the studies of the networks between genes and miRNAs are relatively rare.

The gene expression modifications between cells are associated with various stress survival levels,^[^ ^30^, ^31^, ^32^ ^]^ however, the reasons for changes in transcriptional levels at the single‐cell transcriptome are still unclear. With the continuous expansion of single‐cell RNA sequencing in plant research, the analysis of genes under different stress conditions has increased.^[^ ^33^, ^34^, ^35^ ^]^ In rice, scRNA‐seq has been used to identify major different cell types' responses to abiotic stress, revealing cell‐type heterogeneity in responses to abiotic stress.^[^ ^36^ ^]^ Due to the reliance of most scRNA‐seq technologies on polyadenylated tail priming of messenger RNA (mRNA), they primarily quantify protein‐coding transcripts. Therefore, research on other categories of transcripts, particularly the functional miRNAs, lags behind mRNA studies. Although there have been advancements in measuring miRNA expression in single cells, it is limited to assess a small number of miRNAs per cell or restricted to involve a small number of cells.^[^ ^37^, ^38^, ^39^ ^]^

The biogenesis of miRNAs begins with the transcription of primary miRNA (pri‐miRNA). The majority of pri‐miRNAs are polyadenylated.^[^ ^40^ ^]^ As we all know, scRNA‐seq techniques profile the ‐3′ end of RNA transcripts, which carry the poly‐A tail.^[^ ^41^ ^]^ In scRNA‐seq sequencing datasets, the data is often sparse, and the expression levels of pri‐miRNAs are relatively low. Due to the lack of necessary depth of sequencing coverage, pri‐miRNAs are frequently filtered out from data analysis.^[^ ^42^, ^43^ ^]^ In order to solve these, PPMS (Profile Primary MicroRNAs from Single‐cell RNA‐sequencing) workflow^[^ ^44^ ^]^ helps us to attain sufficient read coverage to profile pri‐miRNAs by aggregating classified cells into the respective cell types. It can reduce sparsity and classify individual cell/nucleus into their respective cell types or cellular states.

In this report, we performed a comprehensive functional dissection of miRNAs in response to hormone ABA by the time series processing and a meta‐analysis of network regulation in both bulk and single‐cell levels. First, we built a GRN by combining ABA‐related TF‐target data and miRNA‐target data and used time series RNA‐seq and small RNA‐seq data to make the dynamic GRN, which helped us to identify miRNA‐containing FFLs (M‐FFLs) and crosstalk regulation. Then we combined these results with single‐cell sequence analysis with early, middle, and late time points. Analyzing data revealed that miRNAs can respond rapidly and cell‐type specifically, which may influence target genes with distinct functions. Furthermore, M‐FFLs also had dynamic responses across various cell types and treatment time points. Even their targets play cell‐type specific regulatory roles within the ABA regulatory network. We hypothesized that the regulatory roles and response patterns of miRNAs together with their targets undergo complex dynamic and cell‐type specific changes during the growth and developmental processes of plants.

## 2 Results

### 2.1 Rapid and Dynamic Genome‐Wide Changes in miRNA Profiles after ABA Treatment in *Arabidopsis*

**Figure 1:**
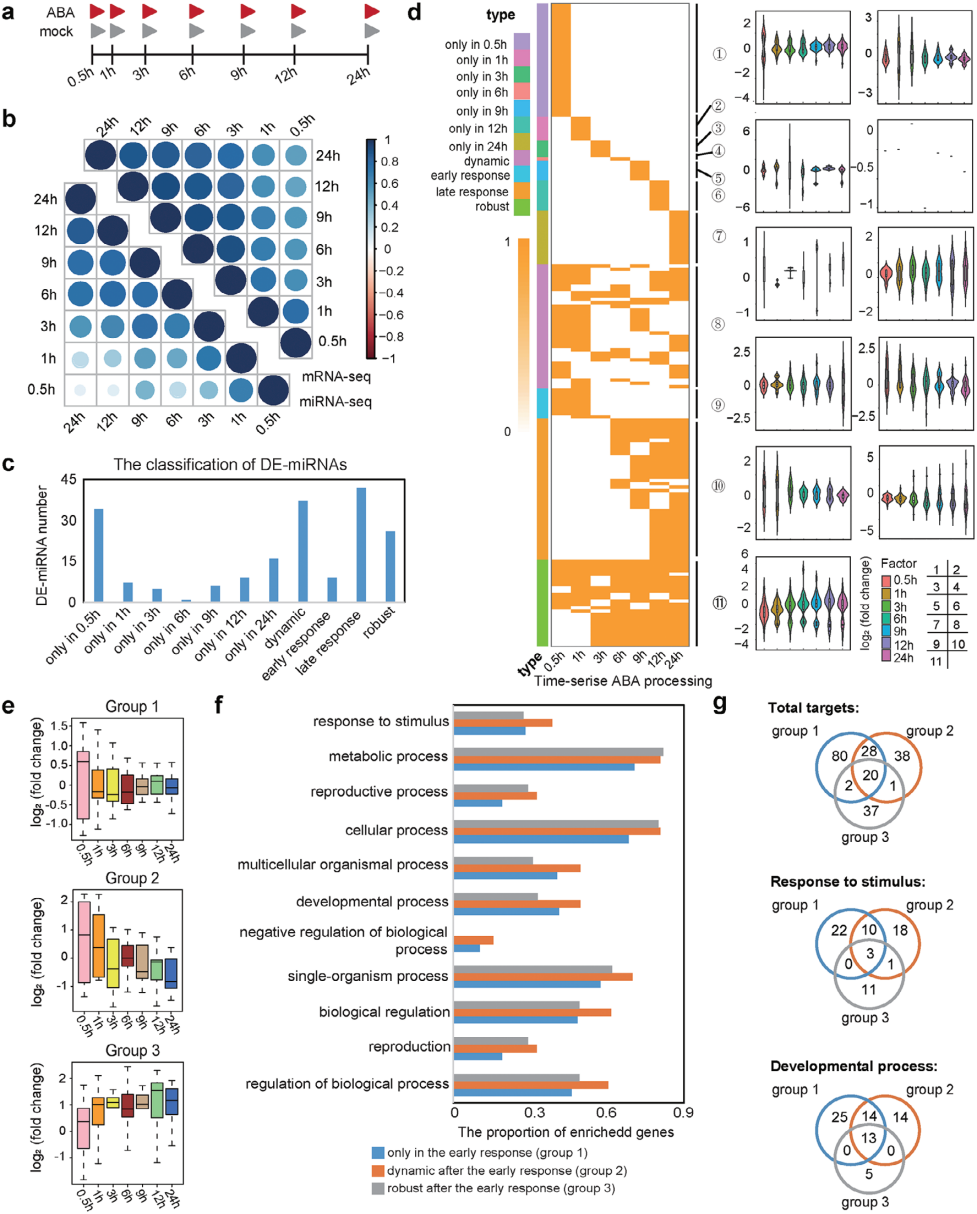
ABA causes genome‐wide changes in miRNA expression. a) The time series processing of *Arabidopsis*: hormone treatment (red) and mock treatment (gray). Hormone treatment is ABA dissolved in ethanol, and mock treatment is the equivalent volume of ethanol. b) Correlation of DEGs (upper right) and DE‐miRNAs (lower left) among the different time points. The changes in miRNAs are more dramatic at the early time points. c) Classification of DE‐miRNAs represents 11 groups of miRNAs: only in 0.5 h, only in 1 h, only in 3 h, only in 6 h, only in 9 h, only in 12 h, only in 24 h, early response, late response, dynamic, and robust. d) Heatmap of the 11 clusters of DE‐miRNAs (left) over the seven time points, and the expression violin plot for each cluster (right). The expression patterns in each cluster differentiated well. e) Three groups of early response miRNAs. MiRNAs in group 1 are only differentially expressed in the early time point. MiRNAs in group 2 have obvious changes in the early time points and have dynamic patterns in the other time points. MiRNAs in group 3 are differentially expressed at most of the time points. f) The enriched GO terms for all the early response DE‐miRNAs’ targets. The X‐axis presents the proportion of enriched genes. g) The comparison among the three groups of early response miRNAs.

We profiled the dynamic transcriptional and post‐transcriptional regulatory networks using time series RNA‐seq and small RNA‐seq of the ABA response at 0.5, 1, 3, 6, 9, 12, and 24 h post‐treatment (**Figure** **1**a). Good repeatability for RNA‐seq and small RNA‐seq was shown by principal components analysis (PCA) (Figure S1a–d, Supporting Information). Length analysis for small RNA‐seq showed enrichment of 21‐nt and 24‐nt peaks, indicating good quality data (Figure S1e,f, Supporting Information). We first surveyed ABA‐responsive genes and miRNAs in *Arabidopsis* at 0.5, 1, 3, 6, 9, 12, and 24 h post‐treatment (Figure 1a). Among the time series RNA‐seq data, 7198 genes were differentially expressed (DE) (false discovery rate (FDR) < 0.05) (Dataset S1, Supporting Information) for at least one‐time point. There were 191 DE‐miRNAs among the same time series of small RNA‐seq data (Dataset S2, Supporting Information).

Following ABA treatment, the regulatory response was dynamic over time (Figure 1b). The correlation between different time points only gradually varied for the DEGs. On the contrary, the correlation between different time points was disordered for the DE‐miRNAs, especially at the early response time points of 0.5 h and 1 h. The other time points for the DE‐miRNAs had a weak correlation with these two early time points, after which, the pattern was similar to that of the DEGs (Figure 1b). The number of DE‐miRNAs gradually decreased over the first three time points, and subsequently increased over the next four time points (Figure S2a, Supporting Information); however, the number of DEGs gradually increased over all the time points (Figure S2b, Supporting Information). These results revealed that the changes in miRNAs were more dynamic than those in DEGs during the early hormone response.

Following Venn's analysis of the seven‐time points, the DE‐miRNAs were divided into 11 categories: only in 0.5 h, only in 1 h, only in 3 h, only in 6 h, only in 9 h, only in 12 h, only in 24 h, dynamic, early response, late response, and robust (Figure 1c; Figure S2a, Supporting Information). The results showed that the vast majority of the DEGs were robust (Figure S2b–d, Supporting Information). The heatmap and the results of the expression analysis of the 11 DE‐miRNA classifications showed the state at various time points (Figure 1d; Dataset S3, Supporting Information). For the robust miRNAs, the fold changes occurred over a large range, while, for the dynamic miRNAs, the fold changes occurred over a narrow range, suggesting that inconspicuous fold changes in expression allow more dynamic variation. These observations suggested that the expression of miRNAs is more rapid and dynamic than that of genes following hormone treatment.

To prove the validity of our data (Figure S3, Supporting Information), for example, following ABA treatment, *miR159* has been shown to increase during seed germination;^[^ ^45^ ^]^ a 1‐day‐old seedling was processed, and the first increase was seen at 4 h. In our study, the first increase in *miR159* appeared at 3 h, with a significant change at 12 h. The response of *miR398* to ABA is dynamic but different between poplar and *Arabidopsis*.^[^ ^46^ ^]^ In our work, the expression pattern of miR398 was also dynamic. *MiR393* increases following ABA stress and the development of lateral roots is inhibited by decreased levels of *TIR1* and *ABF2*, which are *miR393* target genes.^[^ ^47^ ^]^ The powerful role of *miR393* is consistent with the expression pattern of *miR393* found in our study, which belongs to the robust group. Following ABA treatment, expression of *AT5G28520* was increased, and that of *miR846* was decreased. *AT5G28520*, one of the mannose‐binding lectin superfamily protein related to salt and osmotic responses, is the target gene of *miR846*, and their regulation is obvious at 24 h,^[^ ^48^ ^]^ which is consistent with our findings that *miR846* belongs to the late‐response group. Following ABA treatment, the expression pattern of *miR172* was basically the same as that previously described; however, their processing time was longer by at least 24 h.^[^ ^49^ ^]^ Previous small RNA blot analysis of *miR399f* showed dynamic expression before 12 h under ABA processing,^[^ ^50^ ^]^ which is in accordance with our classification of *miR399f* as early‐response. Therefore, our data is consistent with the changes of miRNAs in *Arabidopsis* treated with ABA in the previous reports.

### 2.2 Rapid Response miRNAs Regulate Different Terms of Target Genes at Different Stages

In comparison with the set of miRNA targets obtained in our previous work, the expression consistency between miRNA and its target showed that early‐response miRNAs at 0.5 h and 1 h also had a negative correlation with the late‐response targets (Figure S4a, Supporting Information). The similarity between these time series DE‐miRNAs’ targets showed the same response pattern, which contained the early response, intermediate state, and late response (Figure S4b, Supporting Information).

The miRNAs that began to show differential changes in the early stages were divided into three groups according to the expression patterns in the further time point (Figure 1e). The group 1 miRNAs were only responded in the early response; the group 2 miRNAs had dynamic changes after the early response; the group 3 miRNAs had robust changes after the early response (Figure 1e; Dataset S4, Supporting Information). Although the early response miRNAs had differentiations in the later time points, the biological functions of those miRNAs’ targets were similar, including response to stimulus, developmental process, metabolic process, the cellular process, and other regulation processes (Figure 1f). And most of the targets of miRNAs in each early response groups were diverging (Figure 1g). The targets in group 1 and group 2 were a little similar, and the targets in group 3 were relatively independent of group 1 and group 2. These results confirmed that the early response miRNAs with different expression patterns can influence discrepant target genes.

To further study the biological functions, we analyzed the miRNAs at specific time points found as mentioned above (Figure 1c,d). The expression consistency between miRNA and its targets showed that miRNAs in the specific time point also had a negative correlation only in the early response (Figure S5a, Supporting Information). Based on the target analysis for the early response miRNAs, we compared the targets for specific miRNAs’ sets. These target genes showed more obvious grouping according to the miRNAs’ grouping (Figure S5b–d, Supporting Information). Thus, miRNAs that responded at different stages showed greater differentiation in the regulated target gene sets, especially the miRNAs that responded in the early stages and those in the specific time points.

### 2.3 MiRNAs in the ABA Dynamic Regulatory Network

**Figure 2:**
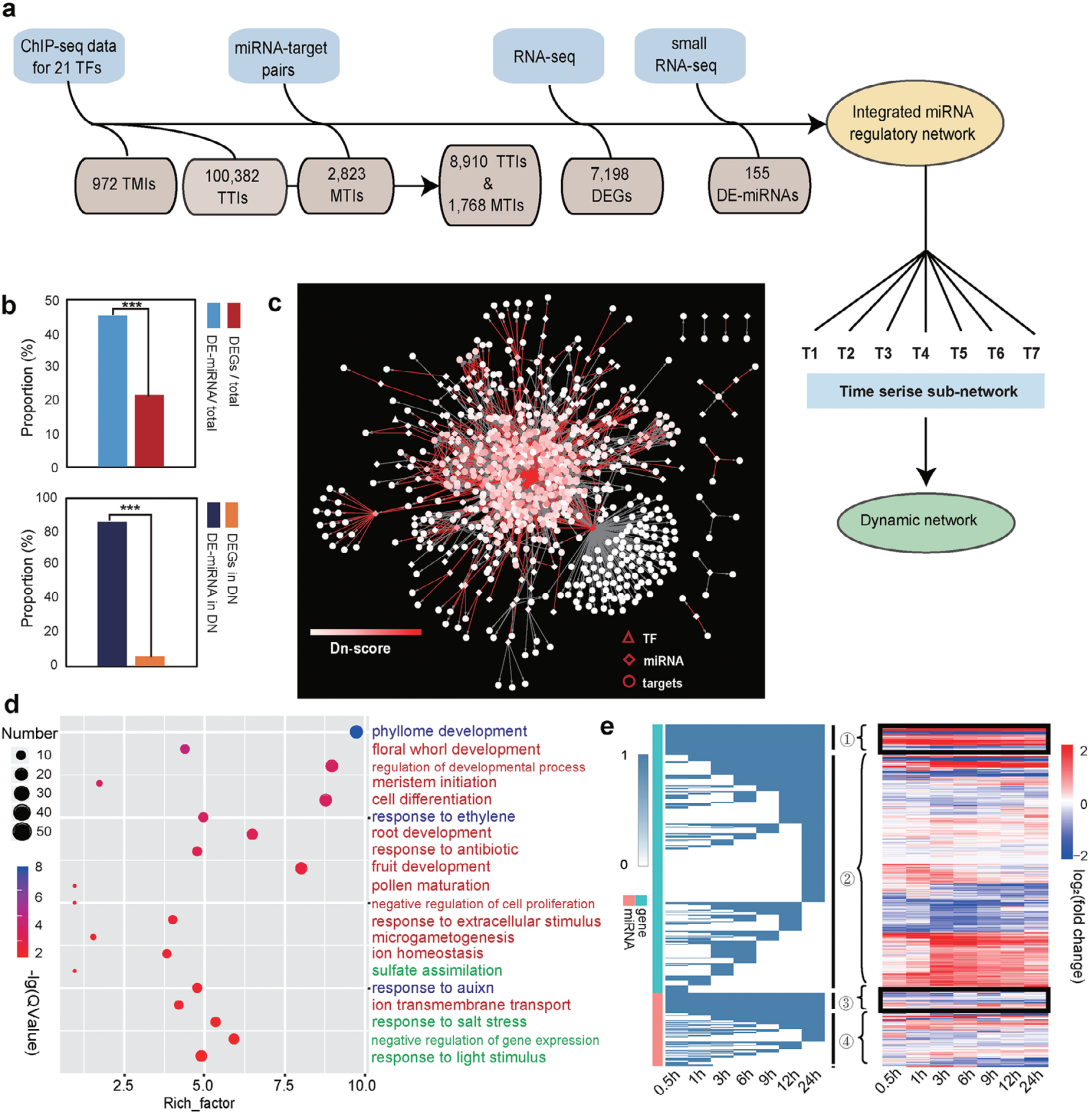
MiRNA's dynamic regulatory characteristics in a dynamic network. a) The source of data used to build the integrated miRNA regulatory network and the formation of the dynamic network. b) The upper panel shows the proportion of differentially expressed miRNAs and genes among the total. The lower panel shows the proportion of DE‐miRNAs and DEGs retained in the final dynamic network. c) Visualization of the dynamic network. Different shapes represent different types of nodes; triangle, TF; rhombus, miRNAs; circle, targets. The color of each node and edge represents the degree of the Dn‐score, which is an important index for dynamic networks. A total of 21 TFs, 189 miRNAs, and 669 targets are included. d) GO term analysis of the target genes in the integrated dynamic network. Red presents the dynamic nodes’ function. Green presents the robust nodes’ function. Blue presents the common function for these two types of nodes. e) Clustering analysis of the dynamic nodes, which contain two clusters of genes and two clusters of miRNAs. The right panel shows the expression pattern of the four clusters.

In order to better study the role of miRNAs in time series post‐stress regulatory pathways, we incorporated multiple sources to reconstruct an integrated miRNA regulatory network for the ABA treatment in *Arabidopsis*. The method for the static network construction was the same as that in our previous work.^[^ ^18^ ^]^ However, the new dynamic regulatory network contains information regarding the DEGs and DE‐miRNAs’ (**Figure** **2**a; Figure S6a,b, Supporting Information). The pattern of TF‐target interactions (TTIs) (Figure S7a,b, Supporting Information) and TF‐miRNA interactions (TMIs) (Figure S7c,d, Supporting Information) in the raw and filter networks were similar. The proportion of DE‐miRNAs in the total miRNAs was significantly higher than that of DEGs (Figure 2b upper). Moreover, the proportion of DE‐miRNAs retained in the final dynamic network was also significantly higher than that of DEGs (Figure 2b bottom). The Dn‐score is an important index for the dynamic network, which favors high‐degree nodes or hubs.^[^ ^51^ ^]^ Nodes with different Dn‐score showed the dynamic characteristics in the network (Figure 2c). Statistical analysis of the nodes in the dynamic network found the nodes with the top 100 Dn‐scores (Figure S8a,b; Dataset S5, Supporting Information). The proportion of dynamic miRNAs was higher than that of dynamic genes among the nodes with the top 100 Dn‐scores, indicating the flexibility of miRNAs (Figure S8a, Supporting Information). In these active nodes with high Dn‐scores, there were miR398^[^ ^46^ ^]^ and miRNA399,^[^ ^50^ ^]^ which flexible functions during the ABA response were introduced earlier in this paper.

Gene Ontology (GO) analysis revealed that the dynamic and robust nodes in the dynamic network had distinct functions. The robust targets are inclined toward the stress response, while the dynamic targets are inclined toward development (Figure 2d; Figure S8c,d, Supporting Information). The core nodes with the top 100 Dn‐scores were enriched in terms related to the response to abscisic acid, secondary metabolic processes, and plant hormone signal transduction (Figure S8b, Supporting Information). And we noticed that most of the nodes were dynamically expressed in the seven‐time points, but the robust nodes had higher expression levels than dynamic ones (Figure 2e). High expression was very important for the stable existence of genes or miRNAs in time series changes. At the same time, low expression could make genes or miRNAs change rapidly at different time points and produce dynamic regulation effects.

The edges in the dynamic network also had distinct variations among the seven‐time points (Figure S9a, Supporting Information). The statistics for MTIs (miRNA‐target interactions) showed that the relationship between miRNA and gene was more single‐minded. Different from one‐to‐many patterns of TFs, most miRNAs had only a small number of target genes, while most of the genes that were targets of miRNAs had only one miRNA bound (Figure S6c, Supporting Information). This characteristic of miRNA binding was related to the dynamic characteristic of miRNAs. Further analysis of miRNA targets showed that the proportion of those dynamic miRNAs was higher than those of robust miRNAs (Figure S9b,c, Supporting Information).

To define the biological function of dynamic miRNAs during the hormone response, we performed an iDREM (Interactive Dynamic Regulatory Events Miner) analysis using the nodes in the dynamic network. The miRNAs were used as an interactive factor in the regulation of targets. The targets were separated into three paths: two increased paths and one decreased path (Figure S9d, Supporting Information). The different sets of dynamic miRNAs contributed to different paths with diverse functions (Figure S9e, Supporting Information). Thus, the sub‐path of dynamic miRNAs also had a specific function. In many important pathways, such as stimulation response, development, regulation, and metabolic process, the common target genes showed more significant enrichment than that regulated by TF alone (Figure S6d, Supporting Information), suggesting that miRNA enhances the regulation of TF‐target genes.

### 2.4 MiRNA Nodes in the M‐FFLs Preferentially Play the Role of Moderator

**Figure 3:**
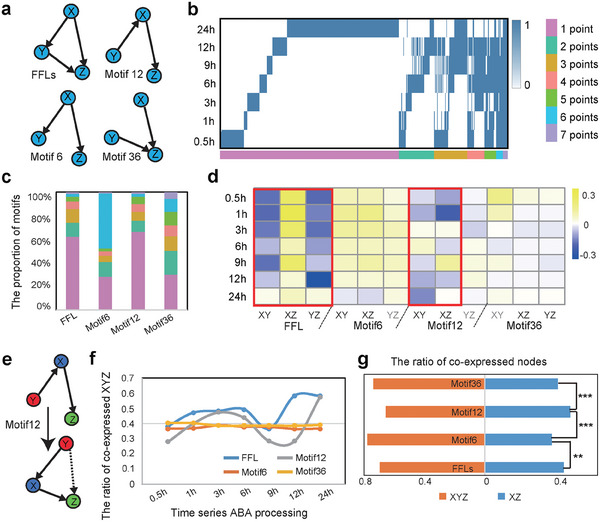
MiRNA nodes in the YM‐FFLs preferentially strengthen the co‐expression pattern in the time series network. a) The formation of four motifs appeared in the dynamic network. b) Classification of FFLs appearing at different time points. When FFL appears at a specific time point, the value is 1, and when it does not appear, the value is 0. The color bar represents the number of time points at which FFL appeared. c) The distribution of the classification of FFL, Motif 6, Motif 12, and Motif 36. The color bar represents the number of time points at which FFL appeared. d) The correlation between every two nodes of the motifs in the network. The grids in the red box are highly correlated. The gray text represents two nodes without arrows in the motif modules. e) Schematic of Motif 12 and rotated Motif 12. f) The ratio of time series co‐expressed XYZ in the motifs. Different colors represent the four motifs. g) The average ratio of co‐expressed XYZ in the four motifs. Orange, co‐expressed XYZ; blue, co‐expressed XZ. ^***^ *p* < 0.001 by the Chi square test; ^**^ *p* < 0.01 by the Chi square test.

In the network, motifs are the fundamental regulatory units. There were four types of motifs in our dynamic network (**Figure** **3**a and **Table** **1**). The FFL had three nodes with three edges that interacted with each two nodes (Figure 3a). As the dynamic feature of our network, we extracted the FFLs according to the seven time‐point networks. According to the Venn analysis of the time series FFLs, most FFLs appeared only in one‐time point, while the least FFLs appeared in all the seven‐time points (Figure 3b), indicating that FFLs were also dynamic.

**Table 1:** Motifs and the statistics in the GRN. The total subgraphs existed in the time series network.

	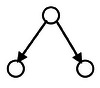	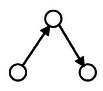	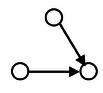	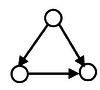	
0.5H	270 768*	1336*	8284*	117	280 505
1H	295 567*	594*	11 077*	65	307 303
3H	372 494*	367*	16 643*	78	389 582
6H	357 145*	747*	15 058*	121	373 071
9H	306 392*	825*	11 890*	123	319 230
12H	311 974*	1327*	12 035*	157	325 493
24H	68 966*	3661*	10 011*	228	82 866

*Note*: The asterisk (*) presents enriched subgraphs comparing with 1000 random network.

In addition to FFLs, there were another three motifs that appeared in our dynamic network: incomplete FFLs with two‐edge interactions (Figure 3a and Table 1). In our network, all the Y nodes in the FFLs were miRNA. The TFs were coordinated by these Y node miRNA‐FFLs (YM‐FFLs) to be located in different hierarchical layers of the regulatory network. In addition, the genes simultaneously targeted by TFs and miRNAs showed a similar expression pattern.^[^ ^52^ ^]^ Other motifs that appeared in our network showed a disordered pattern over time, with the exception of Motif 12, which presented a similar distribution over the seven‐time points (Figure 3c). To mine the regulatory role of miRNAs in the FFL, we statistically analyzed the consistency of expression between every two nodes. The consistency value was high in the FFLs and Motif 12. In contrast, it was low in Motif 6 and Motif 36 (Figure 3d). From the consistency between every two nodes, we found that most of these FFLs in the network were coherent (Figure 3e). The distinction between these two types of motif lies in the function of the miRNA. FFLs and Motif 12 have miRNAs that act like a buffer, which can adjust the direct regulation to be more dynamic.

To further analyze the role of miRNAs, we performed co‐expression analysis using the time series mRNA and miRNA data. There were eight significantly co‐expressed modules of mRNA‐miRNA (Figure S10a, Supporting Information), for which we located the nodes of the motifs. Two co‐expressed forms were found in these four motifs: the X and Z co‐expressed and the X, Y, and Z co‐expressed. The ratio of co‐expressed forms was dynamic in the FFLs and Motif 12; however, that in Motif 6 and Motif 36 was stable (Figure 3f; Figure S10b, Supporting Information). The average ratio of co‐expressed forms in these four motifs showed a distinct difference between miRNA‐mediated and non‐miRNA‐mediated motifs (Figure 3g). These results indicated that miRNA within motifs had more dynamic changes over time.

### 2.5 MiRNAs Link Multiple Intertwined FFLs to make a Crosstalk Regulatory Network

**Figure 4:**
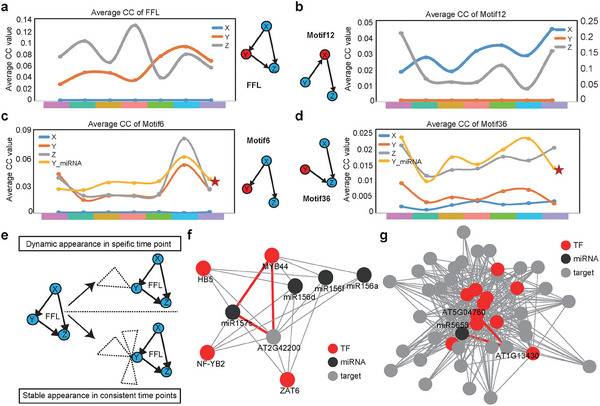
MiRNAs in YM‐FFLs can promote crosstalk regulation in the network. a) The average CC value for X, Y, and Z in FFL. Dark blue, X; orange, Y; gray, Z. The seven color blocks on the X‐axis represent seven types of motifs that appear at different time points. The meanings for X‐axis are the same in Figure 3a. b) The average CC value for X, Y, and Z in Motif 12. c) The average CC value for X, Y, and Z in Motif 6. The CC values for miRNAs in the Y node are calculated separately. d) The average CC value for X, Y, and Z in Motif 36. The CC values for miRNAs in the Y node are calculated separately. e) The model of crosstalk regulation of FFLs in the network. f) An example of miRNA‐mediated FFL appearance at one time point. g) An example of miRNA‐mediated FFL appearance at seven time points.

To gain further insight into the miRNAs within the network, we monitored the topological structure of the motifs. Since crosstalk between FFLs can promote dynamic regulation in the network,^[^ ^53^ ^]^ the average clustering coefficient (CC) value was calculated for each node in the motifs. The clustering coefficient (CC) value is an index that represents the degree of nodes in the network to form the network structure.^[^ ^54^ ^]^ In the FFL, the average CC of the Y node increased according to the number of time points at which it appeared (**Figure** **4**a). However, the other two nodes did not display this trend. As a comparison, we also calculated this score in the other three motifs. There was a similar trend for the X node in Motif 12 (Figure 4b), the miRNA contained in the Y node in Motif 6 (Figure 4c), and the miRNA contained in the Y node in Motif 36 (Figure 4d).

These results demonstrate that the fewer the FFLs connected to a specific FFL, the fewer time points at which the FFL will appear. On the contrary, the more the FFLs connected to a specific FFL, the more time points at which the FFL will appear (Figure 4e). In time series data, the topological rule of FFLs in the network structure is closely related to the regulation of biological function. As time passes, different biological functions are constantly stimulated; accordingly, the more crosstalk between FFLs with different functions, the more active this particular FFL will be since there are different active units at different time points to regulate this particular FFL. For example, an FFL that dynamically appeared (*MYB44‐miR157c‐AT2G42200*) at a specific time point only cross‐talked with 7 other FFLs (Figure 4f). However, an FFL that stably appeared at consistent time points had plenty of crosstalk with other FFLs (Figure 4g). These results suggested that the degree of crosstalk with FFL would affect the regulation range of FFL in time series data.

### 2.6 Cell‐Type Assignment and pri‐miRNA Identification using PPMS on ABA scRNA‐seq Datasets

**Figure 5:**
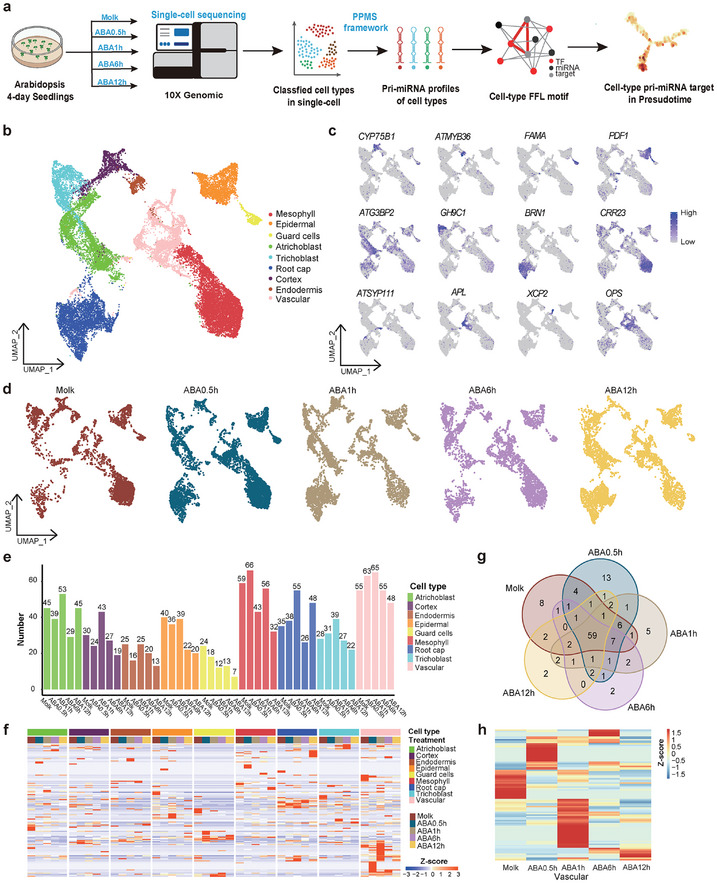
Rapid and cell‐type specific response of pri‐miRNAs during dynamic ABA treatment at the single‐cell level. a) The scRNA‐seq workflow to find out the cell‐type specific pri‐miRNA and targets in dynamic ABA Network. b) Visualization of a total of 9 cell‐type clusters using UMAP. Dots, individual cells; *n* = 25444 cells; color, cell‐type clusters. c) The expression of 9 cell‐type specific genes in *Arabidopsis* seedlings identified in the previous single‐cell transcriptome analysis (*CYP75B1*‐cortex;^[^ ^60^ ^]^ *ATMYB36*‐endodermis;^[^ ^61^ ^]^ *FAMA*‐guard cell;^[^ ^65^ ^]^ *PDF1*‐ epidermal cell;^[^ ^64^ ^]^ *ATG3BP1*‐ atrichoblast;^[^ ^58^ ^]^ *GH9C1*‐trichoblast;^[^ ^58^ ^]^ *BRN1*‐root cap;^[^ ^55^ ^]^ *CRR23*‐mesophyll;^[^ ^62^ ^]^ *ATSYP111*‐vascular_profiling cell;^[^ ^113^ ^]^ *APL*‐vascular_phloem;^[^ ^67^ ^]^ *MYB46*‐vascular_xylem;^[^ ^60^ ^]^ *OPS*‐vascular_procambium).^[^ ^62^ ^]^ The full names of selected genes are given in Dataset S8 (Supporting Information). d) UMAP visualization of seedling samples in five ABA treatment conditions: Mock (no treatment), ABA0.5h treatment, ABA1h treatment, ABA6h treatment, and ABA12h treatment. Different colors represent different ABA treatment conditions. e) Barplot shows pri‐miRNA numbers that are expressed on the different ABA treatments in 9 cell types. f) Heatmap shows pri‐miRNA gene expression that is expressed on the different ABA treatments in 9 cell types. g) The Venn plot summarizes differential pri‐miRNA under different ABA treatment conditions. h) The early response of each pri‐miRNA in the different ABA treatments vascular cells, the changes are more dramatic at the early time points.

ScRNA‐seq provides new insights into studying the cell type's specific responses to the environment.^[^ ^43^ ^]^ The single‐cell transcriptome‐level responses of 4‐day‐old *A.thaliana* seedlings to ABA were investigated at four‐time points: 0.5, 1, 6, and 12 h after ABA treatment (**Figure** **5**a). ScRNA‐seq libraries were constructed and sequenced using the Illumina NovaSeq 6000 Sequencer. The data underwent pre‐filtering at both the cell and gene levels, yielding a dataset comprising over 10 000 cells and ≈23 000 genes in each sample for subsequent analysis (Dataset S6, Supporting Information). Considering the limitations of single‐cell sequencing in detecting miRNA, we profiled single‐cell pri‐miRNAs with PPMS framework.^[^ ^44^ ^]^

Individual cells were tagged using the 10 × Genomics barcode technology, enabling the acquisition of transcriptome data (Dataset S6, Supporting Information). A strong correlation (Spearmen's r > 0.74, p < 2.2e‐16) between gene expression levels obtained through bulk RNA‐seq and scRNA‐seq underscored the high quality of the dataset (Figure S11a–e, Supporting Information). Considering the impact of protoplast isolation on gene expression levels in *Arabidopsis*, we classified genes as “protoplasting‐sensitive” when they demonstrated consistent differential expression (|log2 (fold change)| > 3,q < 0.05) before and after protoplasting (Dataset S7, Supporting Information). The expression data for the protoplasting‐sensitive genes was subsequently omitted from the downstream analysis. Furthermore, we mitigated the influence of the mitochondrial (mito), chloroplast (pt), and ribosomal (ribo) transcriptomes on cell clustering. Finally, a merged dataset comprising five samples was used for subsequent cell clustering (Figure S11f, Supporting Information). We provided a concise overview of the data normalization and integration pipeline, which is elaborated upon in Experimental Section. Cells were classified into 27 distinct clusters and visualized within the same 2‐D UMAP space (Figure S11g, Supporting Information).

Following the exclusion of dead and doublets (Figure S11g, Supporting Information), we acquired high‐quality transcriptome data from 25 444 individual cells. This analysis detected 21821 genes, encompassing ≈85% of the coding genes in the *Arabidopsis* genome. Plotting the transcriptomes from the mock and four ABA‐treated datasets by means of the uniform manifold approximation and projection (UMAP) revealed similar cell distribution patterns across these five groups (Figure 5b,d). Nine major cell clusters were annotated with known gene markers,^[^ ^55^, ^56^, ^57^, ^58^, ^59^, ^60^, ^61^, ^62^, ^63^, ^64^, ^65^, ^66^, ^67^, ^68^ ^]^ including atrichoblast cell, cortex, epidermal cell, endodermic cell, guard cell, mesophyll cell, root cap, trichoblast cell, and vascular cell (Figure 5c). Furthermore, we identified several sub‐cell types within the vascular cell (Figure S12, Supporting Information). The full names of the selected genes can be found in Dataset S8 (Supporting Information), while additional marker gene feature plots are provided in Figure S13 (Supporting Information).

### 2.7 Characteristics of Cell‐Type pri‐miRNA Responses under Dynamic ABA Treatment at Single‐Cell Resolution

Pri‐miRNAs are the initial products in the miRNA biogenesis, influencing both the processing of mature miRNAs and their activity in regulating the target genes.^[^ ^69^ ^]^ PmiREN is a functional plant miRNA database, containing a collection of 177 identified pri‐miRNAs in *Arabidopsis* ^[^ ^70^ ^]^ (Dataset S9, Supporting Information). Among these pri‐miRNAs, 130 pri‐miRNAs were successfully detected (with a TPM> 0) in our single‐cell transcriptome datasets. In most cell types, the number of expressed pri‐miRNAs exhibited an increase at the early stages (0.5 and 1 h after ABA treatment), followed by a decline over time (Figure 5e). The Venn analysis revealed that most time point‐specific pri‐miRNAs were expressed at 0.5 h after ABA treatment (Figure 5g). These results together indicated the rapidity of the pri‐miRNA response to ABA. The total detected pri‐miRNAs displayed dynamic expression changes over time across all nine cell types in response to ABA treatment, indicating a rapid and dynamic pri‐miRNA expression response to ABA, consistent with the results of bulk small RNA‐seq above (Figure 5f). The heatmap presented in Figure 5f further unveiled the cell specificity of pri‐miRNA expression in response to ABA. Apart from pri‐miRNAs, we explored the expression pattern of 21 major TFs responding to ABA across these distinct cell types and found a rapid expression changes, but no cell‐type specific response (Figure S14, Supporting Information). The cell‐type specific M‐FFLs were monitored by cell‐type specific miRNAs, not TFs. The vascular cells displayed a more dramatic pri‐miRNA expression pattern in response to ABA when compared to other cell types (Figure 5f). So, we designated the vascular cell as the pivotal cell type in response to ABA treatment. In vascular cells, the majority of dynamic changes of pri‐miRNA occurred immediately after ABA treatment (Figure 5h). Collectively, these findings revealed the dynamic and cell‐specific characteristics of the pri‐miRNA expression in response to ABA.

### 2.8 Dynamic Regulatory Roles of YM‐FFLs in Cell‐Type Networks

**Figure 6:**
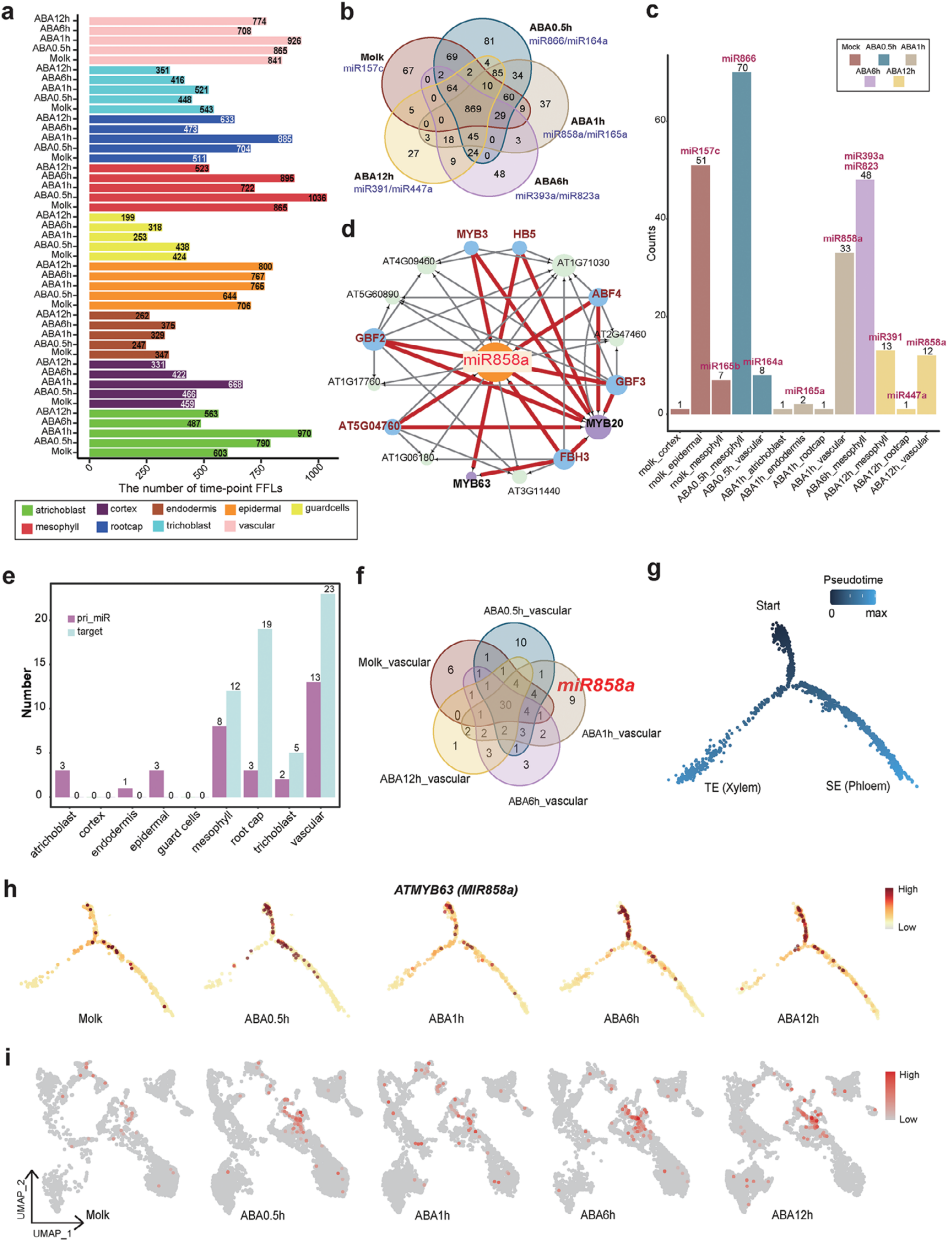
Dynamic and cell‐type specific response changes of pri‐miRNAs and targets in ABA‐related network. a) The distribution of FFLs in each cell type. The color bar represents the percentage of time‐point FFLs that appeared for total motifs. b) The Venn plot summarizes YM‐FFLs in dynamic network under different ABA treatments conditions. The blue highlight shows the cell‐type specific pri‐miRNA in FFLs. c) The cell‐type unique YM‐FFL in dynamic network in 45 groups (5 treatment × 9 cell types). d) The network including miRNA858a in vascular of ABA1h treatment. The red line shows the YM‐FFLs in network, the purple circles are MYB transcription factors, the blue circles are other TFs, the green circles are targets. e) Numbers of cell‐type unique pri‐miRNAs and their targets in dynamic network of 9 cell types. f) The Venn plot summarizes differential pri‐miRNAs under different ABA treatments conditions in vascular, including cell‐type unique pri‐miRNA (miR858a). g) Monocle2 analysis shows vascular differentiation trajectory of TE and SE cells. h) Expression pattern of the MYB63 in different time points along vascular pseudotime. i) UMAP plot shows the expression pattern of MYB63 among different time points in all 9 cell types.

As previously mentioned, motifs serve as the fundamental regulatory units within the network. We generated the ABA response network from single‐cell data, to investigate the regulatory roles of the pri‐miRNA dynamic changes. The network motifs were identified in distinct cell types and four types were observed in every cell type, which was also observed in the previous network (Figure S15a, Supporting Information). Among these motifs, the FFLs displayed the most dramatic changes when responding to ABA, particularly in mesophyll cells, root cap cells, and atrichoblast cells (**Figure** **6**a). In the majority of cell types, the abundance of FFLs was higher during the early stage of ABA response (0.5 and 1 h), consistent with the rapid expression changes of pri‐miRNA. Intriguingly, most of the M‐FFLs specific to different time points included one or two specific miRNAs. For instance, the majority of the M‐FFLs unique in the early ABA response stage contained the miR866, miR164a, miR858a, and miR165a (Figure 6b). Furthermore, the time‐point unique pri‐miRNA also exhibited cell‐type specificity, as the pri‐miR858a was mainly expressed in vascular cells (Figure 6c). These findings implied the regulatory role of a “pioneer” miRNA in specific cell types at various stages of the ABA response.

The pri‐miRNA miR858a was involved in the majority of M‐FFLs in vascular cells, which were the most responsive cells to ABA treatment (Figure 6c,e). Previous studies have unveiled that miR858a exhibited specific activity in vascular tissues during seedling development, and it was found to target a range of regulatory factors, particularly the MYB family, thereby modulating the expression of downstream genes associated with plant development, hormonal and stress responses.^[^ ^71^ ^]^ We found miR858a as a hub node in the ABA response network of vascular cells and could form YM‐FFLs with multiple regulatory TFs and MYB transcription factor family members as its targets (Figure 6d). These findings showed the dynamic regulatory of the miR858a‐mediated network and the potential role of miRNA in the vascular cells to ABA response.

### 2.9 Cell‐Type Specific Roles of miRNAs and their Target Genes in Vascular Tissues

Understanding the cell‐type specific functions of miRNAs and their target genes provides valuable insights into the mechanisms underlying cell identity and tissue differentiation. In our dynamically regulated ABA response network (Figure 2c), the cell‐type‐specific responses were notably distinct. This disparity was particularly pronounced in the mesophyll, root cap, trichoblast, and vascular cells, where the unique pri‐miRNAs and their corresponding targets exhibited significant divergence (Figure 6e). Taken together, miRNAs may exert their plasticity by selectively targeting distinct sets of target genes in various cell types. In vascular cells, it's worth noting that miR858a was present in nearly all unique FFLs at the 1 h time point after ABA treatment (Figure 6f). To gain a deeper understanding of the potential effects of miR858a and its targets across different developmental progress, we performed a pseudotime analysis on vascular cells within annotated cell lineages, and successfully illustrated continuous differentiation trajectories for TEs (xylem) and SEs (phloem) (Figure 6g; Figure S16a,b, Supporting Information).

We have identified miR858a as the “pioneer” miRNA in the early stage of ABA response in vascular cells. A substantial portion of the miR858a target genes belongs to the *Arabidopsis* R2R3 domain‐containing MYB family.^[^ ^71^ ^]^ The MYB transcription factors play a critical role in regulating the formation of secondary cell walls by activating the synthesis of key components such as cellulose, xylan, and lignin. Two MYB family members, MYB63 and MYB20 were identified to be the targets of miR858a specific in the vascular cells (Figure 6h,i; Figure S16c,d, Supporting Information). A previous study has provided evidence that MYB63 serves as the transcription factor responsible for the specific and direct activation of lignin biosynthetic genes. It is worth noting that MYB63 exhibits high expression levels in vascular tissues.^[^ ^72^ ^]^ In our single‐cell data, we observed a dynamic changed expression pattern of MYB63 in vascular cells after ABA treatment (Figure 6h,i). The expression of MYB63 reached its lowest point at 1 h after ABA treatment because miR858a exhibited its highest expression and the most pronounced inhibitory effect on MYB63 at this specific time point. Moreover, MYB63 exhibits an enhanced response to ABA in the cells of the early stage of vascular differentiation, while this effect disappeared in the late differentiation stage cells. Another target of miR858a unique in vascular cells, MYB20, has been reported to activate lignin biosynthesis genes during secondary cell wall formation,^[^ ^73^ ^]^ and also exhibited dynamic expression changes in response to ABA treatment in vascular cells (Figure S16c,d, Supporting Information). The results indicated that the expression of miRNAs and their targets is a highly coordinated process and an alteration of one component may lead to an impediment of another component under abiotic treatment. These results revealed the precise regulatory network in response to ABA, with a nature of temporal specificity and cell‐type specificity.

**Figure 7:**
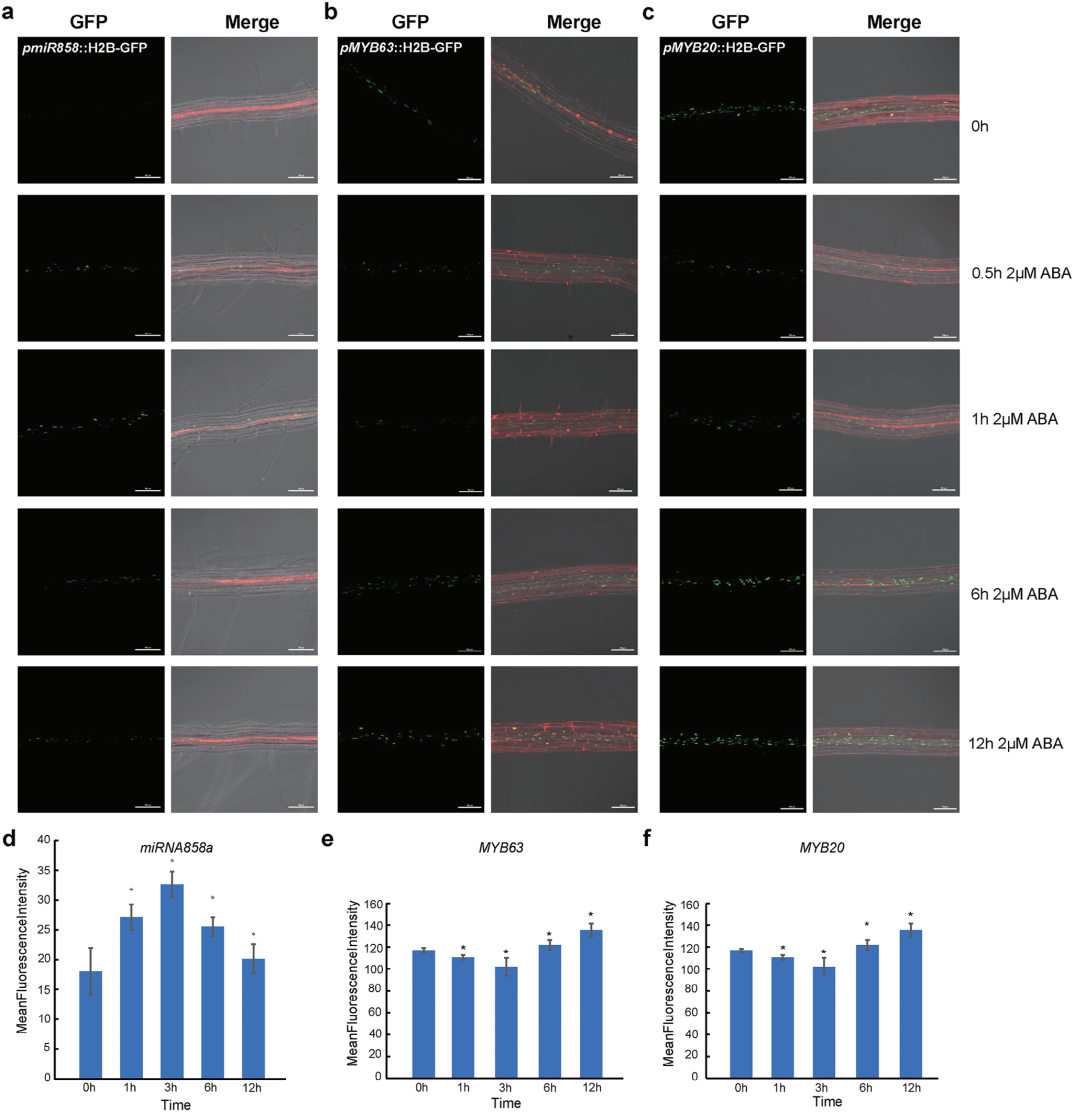
Dynamic changes in the expression pattern of vascular specific *miR858*, *MYB63*, and *MYB20* after ABA treatment. a–c) Dynamic changes in the expression level of *miR858* (a), *MYB63* (b), and *MYB20* (c) in vascular cells. Determine the histone 2B‐GFP (H2B‐GFP, green) reporter driven by thepromoter gene expression pattern using the following method. Under continuous illumination for 4 days, *Arabidopsis* seedlings were incubated with 2 µm ABA for 0.5, 1, 6, and 12 h, and fluorescence expression levels were observed. All images of GFP were captured under the same excitation light intensity. d–f) The average fluorescence intensity of *miR858a* (d, N = 29 roots), *MYB63* (e, N = 20 roots) and *MYB20* (f, N = 19 roots) under 2 µm ABA treatment at different times. The data is represented by the average fluorescence intensity of vascular cells. The asterisk indicates significant differences between treatments (*Tukey test p *< 0.05). The error bar indicates SD.

To investigate the dynamic changes in the expression patterns of target genes for miR858a under ABA treatment, we established the reporter lines for miR858a, MYB63, and MYB20 (**Figure** **7**a–c). In the early stages of ABA treatment, the expression of miR858a is upregulated. This suggests that in the initial phase of ABA treatment, miR858a may play a positive regulatory role in the plant's response to ABA signaling. In the late stages of ABA treatment, the expression of miR858a is downregulated. This may imply that as the duration of ABA treatment extends, the role of miR858a may diminish or change, thereby affecting the plant's long‐term response to ABA. The expression of MYB63 and MYB20 was exactly the opposite of miR858a (Figure 7b,c). Mean Fluorescence Intensity (MFI) is a method for describing the strength of signals from conjugated antibodies, which can be used to semi‐quantitatively analyze the trends in gene expression changes. By measuring MFI, we can visually observe the changes in expression levels of miR858a and its target genes under ABA treatment (Figure 7d–f). In summary, through the validation experiment, we can more visually observe the changes in expression levels of miR858a and its target genes MYB63 and MYB20 under ABA treatment, thereby better understanding their roles in the plant's response to ABA signaling. These findings help us to gain a deeper understanding of the plant's response mechanisms to ABA signaling and may provide new strategies for the genetic improvement of plant stress resistance.

## 3 Discussion

The biological regulation in the organism is a complicated network.^[^ ^25^, ^26^, ^27^ ^]^ The transcriptional and post‐transcriptional regulation presents a dynamic rule under abiotic or biotic responses.^[^ ^29^, ^74^ ^]^ The reported work in plants primarily focused on the static gene regulatory network. Here, we designed time series RNA‐seq, small RNA‐seq, and single‐cell RNA‐seq data to mine the dynamic characteristics of miRNAs. Through the dynamic network, we highlighted the systematical regulatory roles of miRNAs in the function and formation of biological networks and their possible influence in different cells.

### 3.1 The Spatiotemporal Specificity of miRNAs

The most classic characteristics of miRNAs are two aspects–conservation and specificity.^[^ ^1^, ^75^, ^76^ ^]^ Conservativeness refers to the fact that the structure and sequence of miRNAs remain almost unchanged between species, while specificity refers to the temporal and spatial differences in the expression distribution of miRNAs.^[^ ^75^, ^77^ ^]^ In human studies, it has been found that some miRNAs are specifically expressed in different tissues or organs, such as *hsa‐miR‐1* and *hsa‐miR‐133* in muscles, *hsa‐miR‐122* in the liver, and *hsa‐miR‐142* and *hsa‐miR‐181* in the heart. It is found that *hsa‐miR‐122* accounts for 70% expression of all miRNAs in the liver. Further research has found that in cancer cells of the liver, the expression of *hsa‐miR‐122* significantly decreases, while the expression of its target genes increase.^[^ ^78^ ^]^ Therefore, the study of tissue specific miRNAs is of great significance.

Plant miRNAs also have strong specificity.^[^ ^76^, ^77^, ^79^ ^]^ Most miRNAs in *Arabidopsis* are expressed at various developmental stages and organs, with a portion having specific spatiotemporal expression patterns.^[^ ^79^ ^]^ Through high‐throughput sequencing of eight small RNA libraries prepared from diverse abiotic stresses and tissues in *Triticum aestivum*, 257 miRNAs exhibited tissue‐specific expression and 74 were associated with abiotic stresses.^[^ ^77^ ^]^ In the analysis of the spatiotemporal specificity of miRNA in the past two decades, the regulation of proteins and transcription factors upstream of miRNA is still unclear.

In plants, most miRNA specificity studies can be conducted through tissues or time series sequencing, but the results are at the overall level.^[^ ^79^ ^]^ Therefore, combining single‐cell methods with regulatory networks for miRNAs has become a breakthrough to address the spatiotemporal specificity of miRNAs. Mathematical modeling has been used to predict the miRNA regulatory network of single‐cell levels in breast cancer.^[^ ^80^ ^]^ However, as a highly conserved and specific element, miRNAs may have unique characteristics in single‐cell expression, which is different from the models created by genes. Meanwhile, no cell‐type specific miRNA expression or single‐cell regulatory network has been reported in plants. While scRNA‐seq analysis offered insights into the essential cellular activities of miRNAs in plant growth and stress adaptation.^[^ ^81^ ^]^ Therefore, we analyzed the cell‐type specific expression of miRNA by searching for pri‐miRNAs in single‐cell sequencing.

Our results presented new avenues for investigating the cell‐type specific regulation of miRNAs using scRNA‐seq technology. In addition to intrinsic limitations such as low coverage in single‐cell sequencing,^[^ ^44^ ^]^ future advancements in sequencing technologies and algorithms are needed. Further investigation into these cell‐type‐specific regulatory mechanisms promises to shed light on the intricacies of cellular dynamics and functional diversity in biological systems.

### 3.2 Rapid and Dynamic Response of miRNAs under ABA Stress at Single‐Cell Level

MiRNAs have versatile functions in the growth and development of organisms and their response to stress. MiRNAs have very flexible characteristics due to their short length.^[^ ^6^, ^7^, ^82^ ^]^ For a long time, miRNA has been regarded as a transcription regulatory factor that is produced and plays a role in the same cell or adjacent cell groups connected by plasmodesmata. However, the presence of miRNAs in the phloem sap indicates that miRNAs can be transported and act as long‐distance signaling molecules throughout the entire plant.^[^ ^83^, ^84^ ^]^ The movement of miRNAs is the foundation for plant growth and survival. MiRNAs move from cell to cell through intercellular plasmodesmata and coordinate the entire plant's response to stress.^[^ ^85^ ^]^

In our study, we found a rapid response of miRNAs under ABA treatment, which is consistent with the flexibility and mobility of miRNAs. However, our dense time series data showcases the dynamic changes of these miRNAs in detail. Under the stress of ABA, time series sequencing could reflect the dynamic characteristics of expression changes (Figure 1a). The time series transcriptome data showed a trend of gradual change, but the change of miRNAs would be more disordered (Figure 1b). Simultaneously, the ABA0.5h specific DE‐miRNAs accounted for a large proportion (Figure 1c), and there was a strong negative correlation between the early response miRNA and its target gene (Figure S4a, Supporting Information). The same early response was found in sc‐RNA‐seq (Figure 5g). We speculated that miRNAs may be more likely to play a major regulatory role in early response. In other words, miRNAs are more flexible.

**Figure 8:**
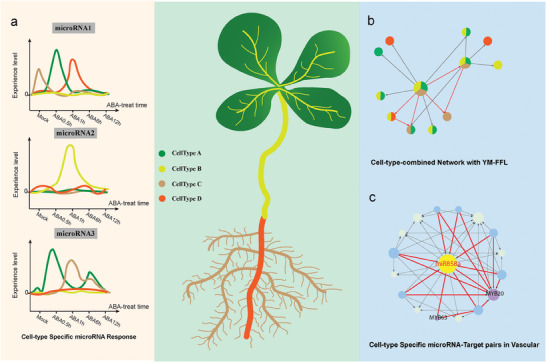
Dynamic and cell‐type specific responses of miRNAs to ABA in *Arabidopsis*. a) The pri‐miRNAs show dynamic and cell‐type specific responses to ABA treatment at single‐cell levels. b) The cell‐type specific regulatory roles of YM‐FFLs within the ABA dynamic regulatory network. c) The *miR858a*‐mediated network including unique YM‐FFL in vascular cells under ABA treatment.

In the previous reports, some miRNAs are responded under ABA, for the exemplar miR159,^[^ ^45^ ^]^ miR398,^[^ ^46^ ^]^ miR393,^[^ ^47^ ^]^ miR846,^[^ ^48^ ^]^ miR172,^[^ ^49^ ^]^ and miR399f.^[^ ^50^ ^]^ Intriguingly, through our accurate cell‐type specific miRNA expression by quantifying the expression of pri‐miRNAs at the single‐cell level, we observed a rapid response of miRNAs to ABA treatment in the majority of *Arabidopsis* cell types, consistent with our bulk data results (Figure 5e). Furthermore, we observed distinct expression patterns of pri‐miRNAs in response to dynamic ABA treatment across different cell types (Figure 5f). We found that the same miRNA may exhibit different patterns of response to ABA in different cell types and time points: miR866 in mesophyll at 0.5 h, miR164a in vascular at 0.5 h, miR858a in vascular at 1 h, miR393a in mesophyll at 6 h, miR447a in root cap at 12 h, and so on (Figure 6c). During the developmental process of the vascular cells (Figure 6f), the targets response to ABA appears to be dynamic and cell‐type specific. Besides, these two MYB factors were uniquely expressed in vascular among all cell types (Figure 6h,i; Figure S16c,d, Supporting Information). In particular, MYB63 exhibits an increased response to ABA in the early progression of vascular differentiation, while this influence diminishes in the later stages. Furthermore, miR858a may have a significant inhibitory effect on the expression of the MYB63 at ABA1h time points, also leading to a noticeable decrease in expression (Figure 6h,i). Overall, specific miRNAs exist in different cell types to play unique roles (**Figure** **8**a).

### 3.3 MiRNAs can Promote the Dynamic and Cell‐Type Specific Regulation by FFLs in Network

The fundamental features of miRNA‐containing FFLs are the intermediated miRNAs in three‐quarters of the FFLs, and miRNAs typically repress the expression of their target genes.^[^ ^18^ ^]^ FFLs are important functional network motifs.^[^ ^86^, ^87^ ^]^ In plants, several FFLs concerning the development process and stress responses have been identified.^[^ ^88^, ^89^, ^90^, ^91^ ^]^ As mentioned above, plant miRNAs were embedded in regulatory networks that coordinated different gene expression programs in support of developmental plasticity.^[^ ^92^ ^]^ For example, developmental transitions were coordinated by the antagonistic activities of *miR156* and *miR172*. While the targets of them also showed complementary patterns.^[^ ^92^ ^]^ Analysis of single‐cell regulatory networks in existing studies of humans refers to the predicted miRNA expression.^[^ ^80^ ^]^ However, there is no relevant analysis in the plant yet.

In order to better study the role of miRNAs in time series post‐stress regulatory pathways, we reconstructed an integrated miRNA regulatory network for the ABA treatment in *Arabidopsis*. In the dynamic network, the YM‐FFLs showed a more dynamic pattern (Figure 3b). Meanwhile, the co‐expression characteristic was more significant with a higher co‐expressed correlation in YM‐FFLs than other motifs (Figure 3f,g). MiRNAs structurally enhanced the FFLs. According to the CC value in the classified FFL statistics, the subtype of M‐FFLs in plants would promote the communications between different functional motifs, so that these motifs showed time series dynamic regulation (Figure 4). In summary, we found that miRNAs could respond rapidly under the stimulation condition, and the overall changes in the early stage were relatively severe. MiRNAs responding at different time points could control the plasticity of miRNA by influencing different terms of target genes in FFLs.

In the motifs identified among all cell types, most proportions of YM‐FFLs are significantly responded at ABA0.5h or ABA1h (Figure 6a). Just like the pri‐miRNA patterns, at the single‐cell level, the response of YM‐FFL to ABA treatment is also variable and cell‐type specific (Figure 6b,c). Previous reports have highlighted the prominent activity of miR858a in the vascular tissue of *Arabidopsis* seedlings, and its involvement in the regulation of flavonoid and lignin biosynthesis modules through the miR858a‐MYB network.^[^ ^69^, ^80^ ^]^ Then we observed a cell‐type specific pri‐miR858a expressed in vascular, and only at ABA1h. In FFL containing network, miR858a was the hub node with two MYB transcription factors, namely MYB20 and MYB63, as exclusive targets of miR858a (Figure 6d). These findings showed the dynamic regulatory and potential roles of the miR858a‐mediated YM‐FFL in the vascular cells.

Meanwhile, the “crosstalk” miRNAs in the YM‐FFLs represent a useful framework for understanding the time series regulation mechanisms. The cell‐type specific regulation in the differentiation increases the dynamic regulation of miRNAs to some extent. Thus, further analysis of the relationships between various kinds of transcriptional and post‐transcriptional regulation and verification of examples can make the dynamic regulation function of miRNAs more clarity (Figure 8b).

### 3.4 Conservation or Divergence in Shared Cell Type?

MicroRNAs (miRNAs) are a class of small non‐coding RNAs that participate in the regulation of gene expression in eukaryotic organisms. The distribution of miRNA families and their members varies greatly among species.^[^ ^93^ ^]^ These differences reflect not only the varying depths of miRNA research in different plants but also the adaptive adjustments of miRNAs during plant evolution. Our analysis of single‐cell data has revealed different expression patterns of miRNAs in various cell types, which may reflect their functions in different biological processes. In different tissues and organs of plants, the regulation of the same miRNA can also differ. Future research needs to better distinguish these cell‐type specific miRNAs in different tissues to better understand their functions and guide subsequent miRNA editing work.

Additionally, subsequent work can analyze the conservation of miRNAs and their target genes across different species through comparative analysis, functional validation, network analysis, and evolutionary analysis to understand their functional changes during the evolutionary process. With the development of gene‐editing technologies, the application of CRISPRi technology in specifically inhibiting miRNA clusters and miRNAs with high sequence homology can help identify the effects of miRNA overexpression in different disease states compared to normal physiological expression.^[^ ^94^ ^]^

In summary, our findings suggest that miRNAs show dynamic and cell‐type specific responses to ABA treatment, with a significant proportion of early responses. Besides, we speculated that miRNA‐contained FFLs play a significant role in rapid and dynamic responses to ABA in the network. Meanwhile, we have discovered certain cell‐type specific miRNAs that can respond to ABA by targeting cell‐type specific target genes during certain cell type development stages (Figure 8).

## 4 Experimental Section

### Plant Materials and Growth Conditions

*Arabidopsis* ecotype *Col‐0* was used in the present study. The *Arabidopsis* was cultivated in MS medium (MS: 4.4G/L; Sucrose: 1%; Agar: 0.8%; PH: 5.8) with full illumination (intensity) and a temperature of 22 °C. The ABA concentration used was 2um. The seedlings were separated into two groups for hormone processing. One group of seedlings was incubated with ABA for 0.5, 1, 3, 6, 9, 12, and 24 h, and simultaneously, the mock‐processed seedlings were curated for the same periods without ABA. At each of these time points, the seedlings were collected for subsequent sequencing.

### Protoplast Isolation

According to 10 × Genomics User Guide, there are strict requirements on protoplast purity and concentration. The seedlings were placed into a 35‐mm‐diameter dish containing a 70‐mm strainer and 5 mL RNase‐free enzyme solution (1.5% cellulase R10, 0.4 m mannitol, 0.1 m 4‐morpholineethanesulfonic acid, 10 mm KCl, 10 mm CaCl2 and 0.1% BSA). The dish was rotated at 70 rpm for 2 h at room temperature, then the protoplasts were filtered 2–3 times with cell strainers (40 mm in diameter, Falcon, Cat No./ID: 352 340). After that, the cell solution was centrifuged at 100 g for 7 min and the pellet was resuspended in 30–50 mL washing solution (0.4 m Mannitol, 20 mm MES (pH 5.7), 20 mm KCl,10 mm CaCl2, 0.1% BSA). The filtered solution was centrifuged 3–4 times at 100 g for 2 min to achieve the desired cell concentration concentrated at room temperature. The protoplast viability was determined by trypan blue staining.

### High Throughput RNA‐Sequencing

Total RNA was extracted using the RNeasy Plant Mini Kit (Qiagen). High‐throughput RNA sequencing was performed using the Illumina HiSeq 2000 platform. Construction of the sequencing library was completed by a sequencing company (GENEWIZ). The sequence length was 2 × 150 bp (paired‐end) for mRNA‐seq, and the output was no less than 6 G data per sample.

The reads generated by RNA‐seq were initially tested by fastQC and processed to remove the low‐quality (Q value < 30) reads and adapter sequences. To obtain the high‐quality reads, the first 9 bp from each read were cut using Perl script, and the remaining bp were mapped to the TAIR10 reference using the Spliced Transcripts Alignment to a Reference (STAR) tool.^[^ ^95^ ^]^ The default parameters were used, which gave mapping rates greater than 90%. Subsequently, the significantly differentially expressed genes were calculated using the Cuffdiff software. The screening criteria for significantly differentially expressed genes were a fold change (FC) ≥ 2 and a Q value ≤ 0.05.

### High Throughput Small RNA‐Sequencing

Total RNA was extracted using the RNeasy Plant Mini Kit (Qiagen). High‐throughput small RNA sequencing was performed using the Illumina NextSeq 500 platform. Construction of the miRNA sequencing library was completed by a sequencing company (BerryGenomics). The sequencing mode was 75SE (single‐end), and each sample produced no less than 25 M clean reads.

The reads generated by RNA‐seq were initially tested by fastQC and processed to remove the low‐quality (Q value < 20) reads and adapter sequences, following which the possible rRNAs were removed using the Bowtie2^[^ ^96^ ^]^ and the distribution of the small RNAs was calculated. Subsequently, the high‐quality reads were mapped to the TAIR10 reference using the Bowtie tool.^[^ ^97^ ^]^ The default parameters were used, with the exception of no mismatch parameter, which gave a mapping rate greater than 83%. The significantly differentially expressed miRNAs were calculated by the HT‐seq^[^ ^98^ ^]^ and DE‐seq.^[^ ^99^ ^]^ The screening criteria for significantly differentially expressed miRNAs were an FC ≥ 1.5 and a *p* value ≤ 0.05.

### Single‐Cell RNA‐Sequencing

According to the manufacturer's introduction, Single‐cell RNA‐seq libraries were constructed using Single Cell 3′ Library and Gel Bead Kit V3.1 The libraries were finally sequenced using an

Illumina Novaseq6000 sequencer with a sequencing depth of at least 100 000 reads per cell with pair‐end 150 bp (PE150) reading strategy (performed by CapitalBio Technology, Beijing).

### Statistics and Basic Analysis of RNA‐Seq and Small RNA‐Seq Data

The consistency analysis of DEGs and DE‐miRNAs between different time points was conducted by R package Hmisc (https://cran.r‐project.org/web/packages/Hmisc/) and Corrplot (https://cran.r‐project.org/web/packages/corrplot/index.html). The principal component analysis (PCA) was conducted by R package Psych (https://cran.r‐project.org/web/packages/psych/index.html). The miRNA targets were identified in our previous work.^[^ ^18^ ^]^ The similarity of genes’ function between the time series gene sets was analyzed by GOSemSim (http://www.bioconductor.org/packages/release/bioc/html/GOSemSim.html). The agriGO v2.0 (http://systemsbiology.cau.edu.cn/agriGOv2/) was used to complete the GO term analysis.^[^ ^100^ ^]^ Venn analysis was conducted by VENNY 2.1 (https://bioinfogp.cnb.csic.es/tools/venny/index.html) and another webtool Venn (http://bioinformatics.psb.ugent.be/webtools/Venn/). The visualization of multiple Venn diagrams could be realized by the R package UpSetR (https://cran.r‐project.org/web/packages/UpSetR/index.html). The expression correlation matrix between miRNA and its target gene was obtained by command rcorr.

The R command cor.test was used to calculate the Pearson correlation coefficient of the fragments per kilobase per millon reads (FPKM) for each gene or miRNA. Statistical analyses were performed using the R command chisq.test and t.test, which were independent‐samples t‐test (t.test(x, y, paired = FALSE)) by R library (car).

### Dynamic Network Construction and Analysis

The TF‐target sets and miRNA‐target sets were obtained from the previous work.^[^ ^18^ ^]^ The static network contained TF‐target interactions (TTIs), miRNA‐target interactions (MTIs), and TF‐miRNA interactions (TMIs). Time series gene expression data made the network dynamic. The dynamic network was conducted using the plug‐in DyNet Analyzer of Cytoscape (version 3.7.2).^[^ ^51^, ^101^ ^]^ The dynamic nodes, edges, and DnScore^[^ ^51^ ^]^ were all collected from DyNet Analyzer.^[^ ^51^ ^]^ Edge‐weighted Spring Embedded Layout method was used to display the general situation of the network.^[^ ^101^ ^]^ NetworkAnalyzer module was used to examine global topology.^[^ ^101^ ^]^

The motifs enriched in the network were found using Mfinder^[^ ^102^ ^]^ by comparing the real network and random network, which had the same number of nodes and edges but randomized connections. To analyze the structure of miRNAs, the clustering coefficient^[^ ^54^ ^]^ for all nodes were determined by Cytoscape.^[^ ^101^ ^]^

The iDREM (Interactive Dynamic Regulatory Events Miner) was used to conduct the pathway expression pattern analyse.^[^ ^103^ ^]^ TF‐targets and miRNA‐targets information were added to the expression regulation and the genes were divided into different paths. Each term of regulators could be extracted from the results of iDREM.

### Co‐Expression Analysis for mRNA‐miRNAs

Co‐expression data was analyzed using WGCNA (Weighted correlation network analysis) tool.^[^ ^104^ ^]^ The time series expressed genes and miRNAs were integrated for co‐expression analysis. First, the abnormal values in each sample were detected, and the genes or miRNAs not expressed in any time point were removed. Then, choosing a β value to establish the proximity matrix, according to the connectivity, The gene distribution could be made to conform to the scale‐free network. Next, getting proximity matrix and topological matrix from β value. Finally, for the topological matrix obtained, dissimilarity between genes was used to cluster genes, and then used dynamic cutting method to cut trees into different modules (the minimum number of genes in modules was 30). 36 modules were retained based on the above analysis, of which eight modules showed significant correlation with stress trait.

### Single‐Cell RNA‐Seq Data Analysis

The raw scRNA‐seq dataset was first analyzed by Cell Ranger 6.1.2 (10 × Genomics). Run “cellranger mkref” arguments to build reference. Run “cellranger count” arguments to generate single‐cell gene counts. The genome and GTF annotation files of *A. thaliana* were TAIR10. Reads were aligned to the TAIR10 reference genome by the aligner STAR.^[^ ^95^ ^]^ The detailed Cell Ranger reports were given in Dataset S4 (Supporting Information). The “filtered_gene_bc_matrices” by 10 × Genomics were served as processed raw data for further analyses.

Doublets in each scRNA‐seq dataset were detected with DoubletFinder (v.2.0.3).^[^ ^105^ ^]^ The number of expected real doublets (nExp), the number of artificial doublets (pN), and the neighborhood size (pK) are required for doublets prediction. For nExp, the standard Seurat processing pipeline was performed with the low cell cluster resolution (resolution = 0.5), and the doublet ratio with N/100 000 (N, the cell numbers). The nExp value was adjusted according to the proportion of homotypic doublets and doublet ratio. pN was set to 0.25. To identify optimal pK value, we loaded the “paramSweep_v3 (PCs = 1:15)” function to pre‐processed Seurat data and subsequently fed into “summarizeSweep” and “find.pK” functions, to the optimal pK parameter. The doublets were finally predicted with the pre‐processed Seurat data using “doubletFinder_V3” function and the defined values of nExp, pN and pK as defined above. Cells which were flagged as singlets were kept for further downstream analysis.

The unique molecular identifier (UMI) count matrix was processed using the Seurat R package (v.4.3.0).^[^ ^106^ ^]^ For quality control, the low‐quality cells and genes were filtered as follows: 1) only the cells in which the number of expressed genes was 500 to 10 000 were considered; 2) the cells with unique molecular identifiers (UMIs) above 70 000 and below 500 were filtered out; 3) the cells with the percentage of mitochondrial genes more than 10% were excluded; 4) the cells with the percentage of chloroplast genes exceeding 10% were removed.

Briefly, gene expression levels were normalized using function “SCTransform” to reduce the influence from variation in the cellular sequencing depth.^[^ ^107^ ^]^ The 3000 most highly variable genes across samples were selected using the function “SelectIntegrationFeatures”. Finally, the reference‐based Seurat integration workflow was employed to integrate multiple samples.^[^ ^108^ ^]^ Molk sample were chosen as the reference. Overall, 5 samples were used to build the atlas.

Linear dimensionality reduction of the integrated scRNA‐seq data was performed using function “RunPCA” in the Seurat R package. For PC analysis, the scaled data were reduced to 100 approximate PCs (set npcs = 100). UMAP dimensional reduction^[^ ^109^ ^]^ was used for 2‐D visualization using 60 principal components. Cells were further clustered according to the top 60 principal components based on the Louvain algorithm, using functions “FindNeighbors” and “FindClusters” (resolution = 1) in the R package Seurat.

### Correlation Analysis of Single‐Cell and Bulk

The FPKM expression values of bulk RNA‐seq were calculated for each gene. The bulk RNA‐seq counts and pseudo‐bulk scRNA‐seq counts were transformed into log2 scale to minimize and normalize the differences in library size. For correlation analysis, the Speardman's rank correlation between bulk RNA‐seq dataset and scRNA‐seq dataset was conducted in R.

### PPMS Workflow

Pri‐miRNAs are less abundant than mRNAs and are often filtered out from data analysis. The PPMS^[^ ^44^ ^]^ framework in R which is available on GitHub (https://github.com/SrivastavaLab‐ICL/PPMS) was used to profile 177 pri‐miRNAs at the single‐cell‐type resolution.

### Pseudotime Analysis of Sub Clusters

Unsupervised ordering of the subcluster was done with the Seurat integrated results as input to build a tree‐like differentiation trajectory using the DDRTree algorithm of the R package Monocle v2.^[^ ^110^ ^]^ “Scaled Expression” indicates batch‐corrected, log‐normalized values extracted from the slot “data” of a Seurat object's “integrated” assay. For the three pseudotime ordering analyses, the 3000 gene expression matrix, scaled and regressed from the Seurat integrated samples was loaded into Monocle2 using the newCellDataSet function (lowerDetectionLimit = 0.1, expressionFamily = uninormal()). The top 1000 var_genes in Seurat were set as ordering genes and trajectory building was made by calling the reduceDimension Monocle function (max_components = 2, reduction_method = “DDRTree”, norm_method = “none”, pseudo_expr = 1). ABA_treattime‐specific genes were subsequently identified with the “≈differentialGeneTest” function. These genes were used as features to reconstruct cell trajectories with Seurat.

### The Binding Position of miRNAs’ Targets and Structure of miRNAs

The position of miRNA target genes could be divided into the following categories: promoter, 5′ UTR, CDS, and 3′ UTR. The binding positions were confirmed by psRNATarget (http://plantgrn.noble.org/psRNATarget/).^[^ ^111^ ^]^ Default parameters were used to conduct psRNATarget analysis.

### Report Line

The seed‐specific AT2S3 promoter is used to drive the expression of DsRed2.^[^ ^112^ ^]^ The promoter of target genes is used to drive the expression of GFP. *Arabidopsis* seedlings grown for 4 days were incubated with 2 µm ABA for 0.5, 1, 6, and 12 h.

### Statistics of Mean Fluorescence Intensity (MFI)

The formula for calculating MFI: Mean Fluorescence Intensity (Mean) = Total Fluorescence Intensity (IntDen)/Area (Area).

## Conflict of Interest

The authors declare no conflict of interest.

## Author Contributions

Z.G, Y.S, G.J, and Z.L. contributed equally to this work. X.W.D. and H.H. conceived and designed the project. Z.G. and Y.S. performed the bioinformatics analyses. Z.G. and Y.S. performed the single‐cell extraction experiments and managed sequencing. Z.L. and X.H. performed the reporter line construction experiments. L.C., C.X., Z.W., and J.L. helped to perform the single‐cell extraction experiments. R.Y. prepared the RNA‐seq and small RNA‐seq materials. X.H. contributed to the single‐cell RNA‐seq analysis discussion. Z.G. and G.J. wrote the manuscript. X.W.D. and H.H. revised and supervised the manuscript.

## Supporting information



Supporting Information

Supporting Information

Supporting Information

Supporting Information

Supporting Information

Supporting Information

Supporting Information

Supporting Information

Supporting Information

Supporting Information

Supporting Information

## Data Availability

RNA‐seq and small RNA‐seq data generated in this article was submitted to the NCBI's Gene Expression Omnibus (GSE145209). The ChIP‐seq data used can be found as GSE80568 in the same data platform. The single‐cell RNA‐seq data is available from the National Center for Biotechnology Information (NCBI) under the BioProject PRJNA941486.
