# Supplementary material for: Cell‐Type Specific miRNA Regulatory Network Responses to ABA Stress Revealed by Time Series Transcriptional Atlases in Arabidopsis
Source: Adv Sci (Weinh). 2025 Jan 10;12(9):2415083. doi: 10.1002/advs.202415083 (PMC11884551; doi:10.1002/advs.202415083)
Supplement: Supplementary file 1 — Supporting Information [file ADVS-12-2415083-s001.docx]

**Supplementary material**

**
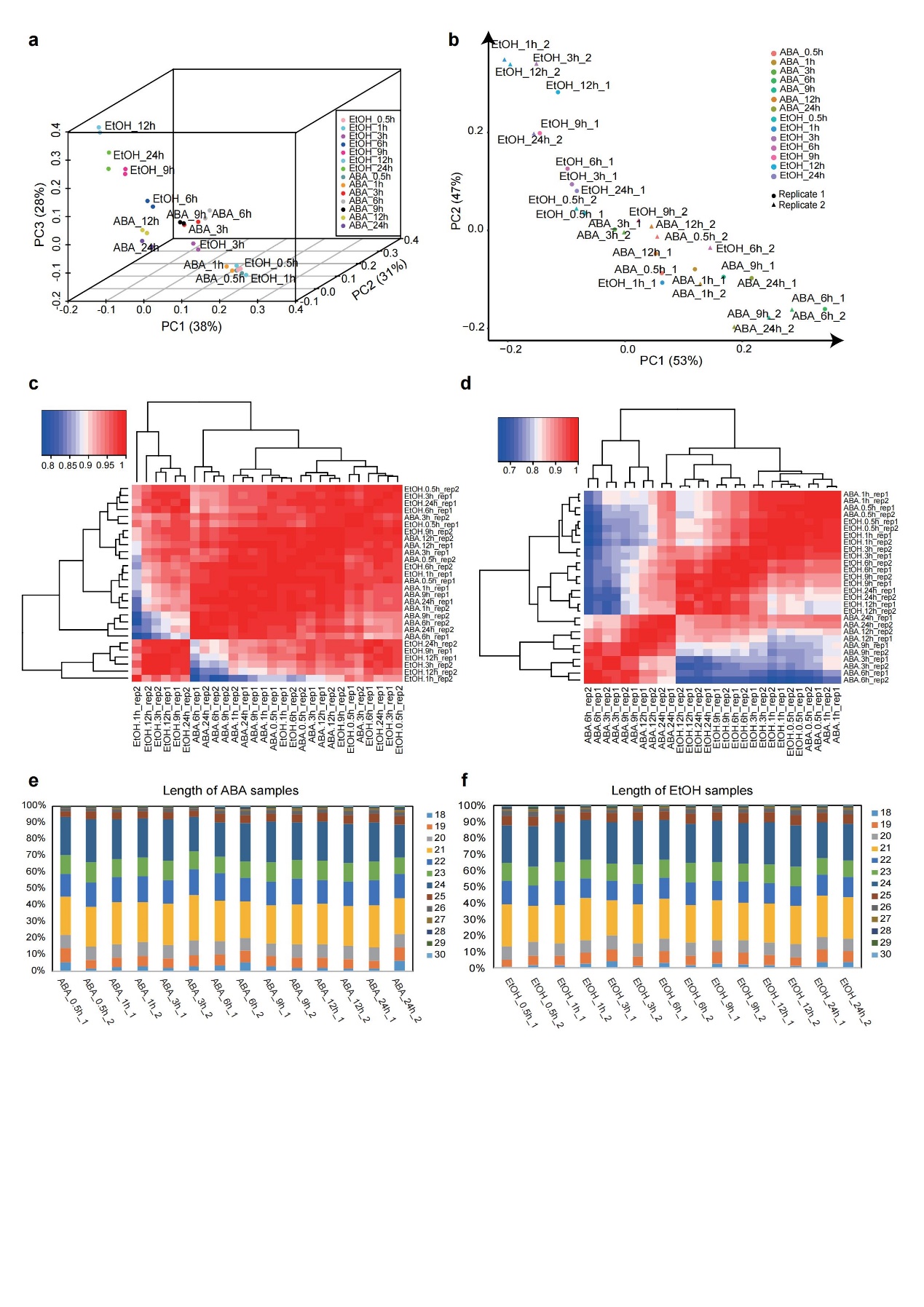
**

**Figure S1. The quality of RNA-seq and small RNA-seq. a** Principal component analysis illustrating the relationships among the mRNA-seq libraries generated from time series data. Different time points are labeled according to the key. Three components can explain 97% of the variation. **b** Principal components analysis illustrating the relationships among the small RNA-seq libraries generated from time series data based on miRNA levels. Different time points are labeled according to the key. Two components can explain 100% of the variation. **c and d** The consistency between different samples for RNA-seq (**c)** and small RNA-seq (**d)**. **e and f,** The length distribution of small RNAs for ABA-treated samples (**e)** and mock samples (**f)**.

**
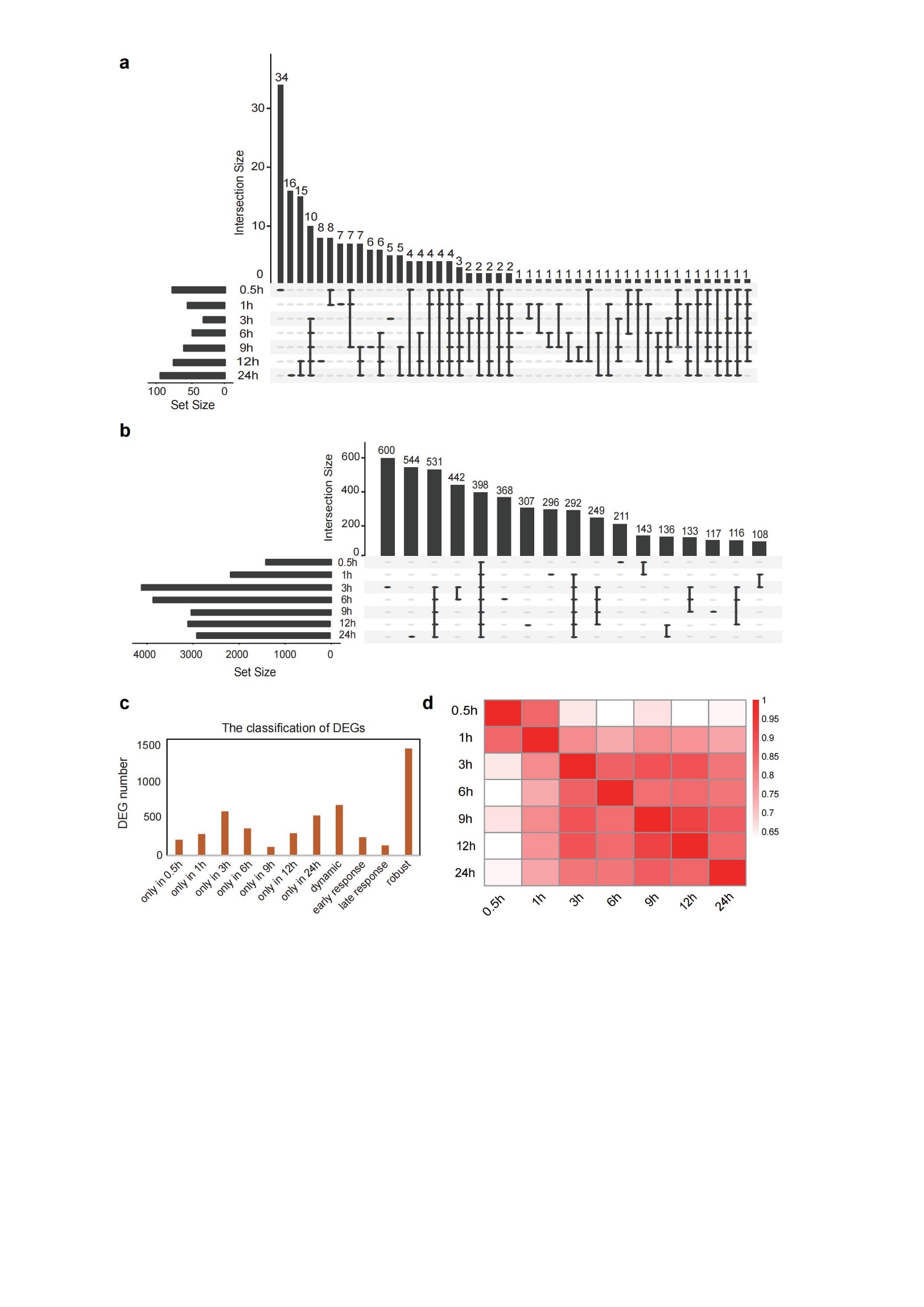
**

**Figure S2. The feature of DEGs in seven time points. a** Upset analysis of DE-miRNAs showing the expression features of miRNAs over the seven time points. **b** Upset analysis of the 7198 DEGs over the seven time points. **c** Classification of DEGs presenting 11 groups of miRNAs, at 0.5 h, at 1 h, at 3 h, at 6 h, at 9 h, at 12 h, at 24 h, early response, late response, dynamic, and robust. **d** Correlation analysis of the differences in DEGs between two time points using GoSemSim.

**
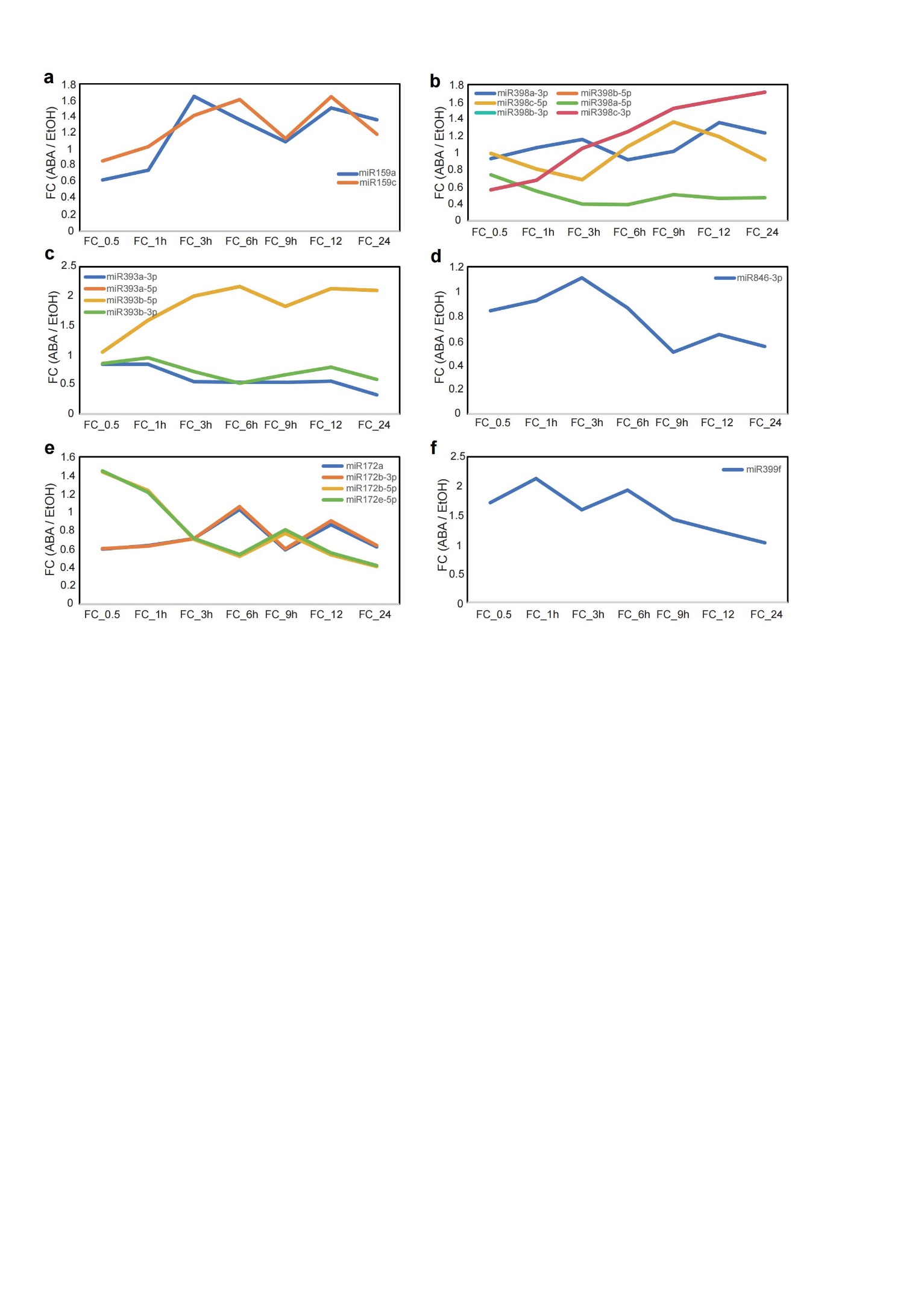
**

**Figure S3. The expression patterns for miRNAs in our time series transcriptome analysis. a** is for miR159. **b** is for miR398. **c** is for miR393. **d** is for miR846. **e** is for miR172. **f** is for miR399.

**
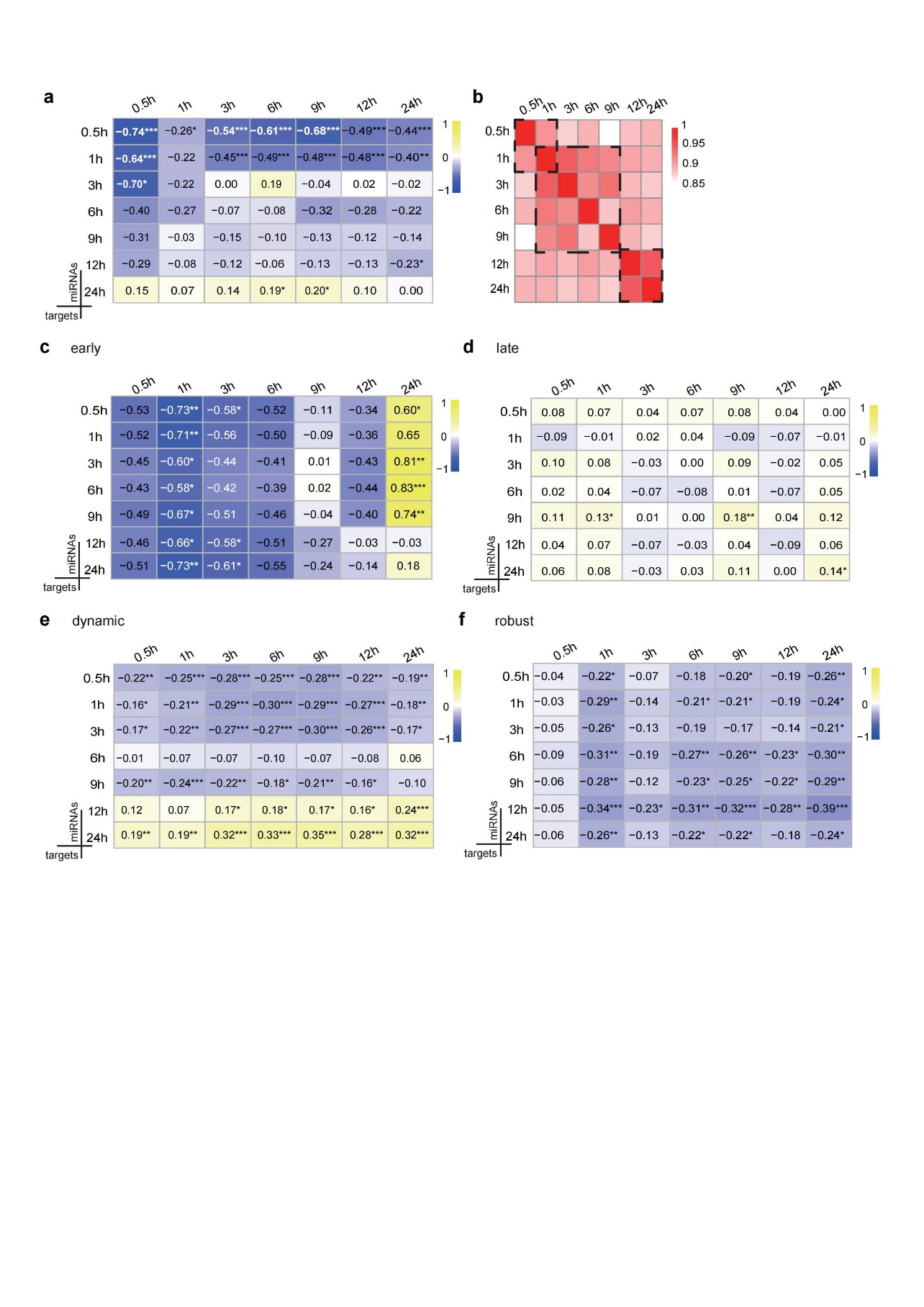
**

**Figure S4. The expression consistency between DE-miRNAs and DE-miRNAs’ targets. a** The expression consistency between DE-miRNAs and their differentially expressed targets. The Y-axis shows DE-miRNAs and the X-axis shows DEGs. The asterisk represents significant consistency (*** *P* ≤ 0.001, ** *P* ≤ 0.01, * *P* ≤ 0.05). The DE-miRNAs at 0.5 h and 1 h have a strong correlation with DE-targets at the later time points. **b** Correlation analysis of the difference in DE-miRNA target genes between two-time points using GOSemSim. The black dotted line dividing the similar pattern shows the three transformation processes of the early phase, transition phase, and late phase following hormone treatment. **c** The expression consistency between early-stage DE-miRNAs and DE-miRNAs’ targets. **d** The expression consistency between late-stage DE-miRNAs and DE-miRNAs’ targets. **e** The expression consistency between dynamic time point DE-miRNAs and DE-miRNAs’ targets. **f** The expression consistency between robust time point DE-miRNAs and DE-miRNAs’ targets. The Y-axis shows the DE-miRNAs, and the X-axis shows the DEGs. The asterisk shows significant consistency (*** presents *P* value ≤0.001, ** presents *P* value ≤0.01, * presents *P* value ≤0.05).

**
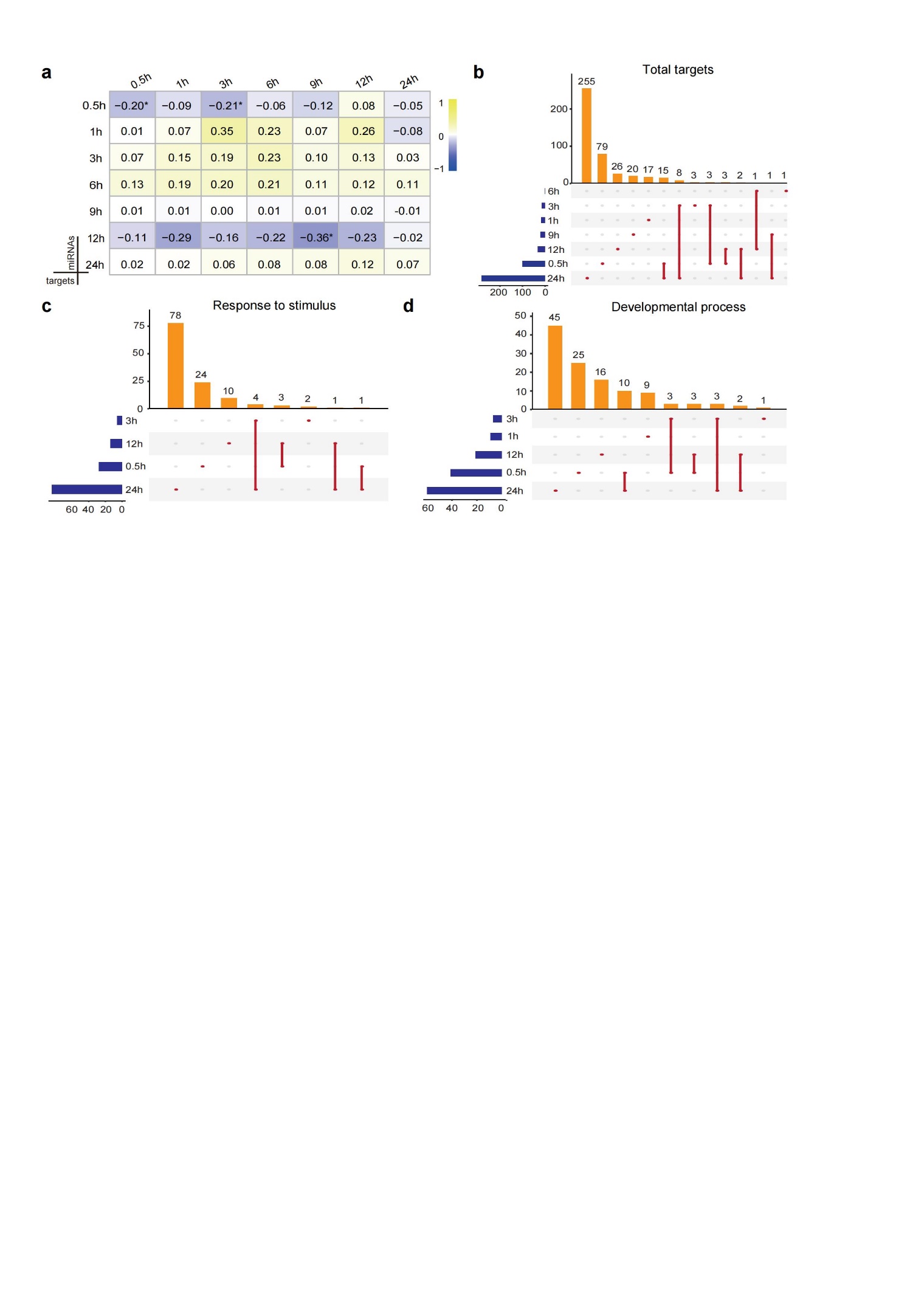
**

**Figure S5.** **The miRNAs responded at specific time points. a** The expression consistency between DE-miRNAs and miRNAs’ targets in specific time points. The Y-axis shows the DE-miRNAs, and the X-axis shows the DEGs. The asterisk represents significant consistency (*** *P* ≤ 0.001, ** *P* ≤ 0.01, * *P* ≤ 0.05). **b-d** The distribution of miRNA target genes in specific time points shows the comparison between each two time points. **b** for the total targets. **c** for the response to stimulus targets. **d** for the development process targets.

**
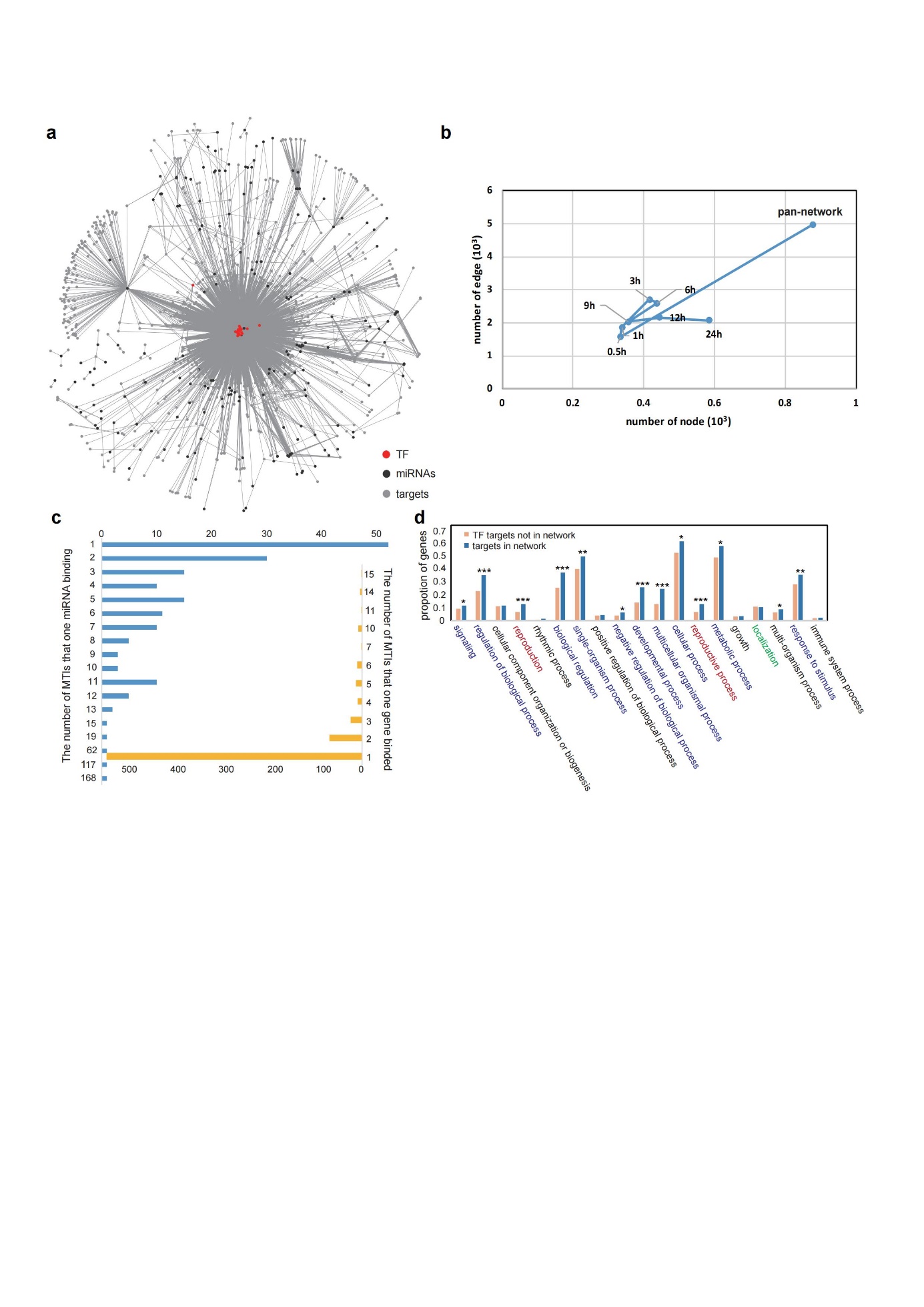
**

**Figure S6. The construction of integrated miRNA regulatory network and dynamic network. a** Visualization of the network containing TFs, miRNAs, and TF miRNA targets. **b** The dynamic course of the time-series network and pan-network. **c** The dynamic features of MTIs in the dynamic network. The upper left shows the number of genes that bound the miRNAs, and the lower right shows the number of miRNAs that bound the genes. **d** The common target genes of TF and miRNA in the network are more significantly enriched in the functional enrichment analysis than other targets that are not in the network. The asterisk shows the significance of the *Chi-square* test (*** *P* ≤ 0.001, ** *P* ≤ 0.01, * *P* ≤ 0.05).

**
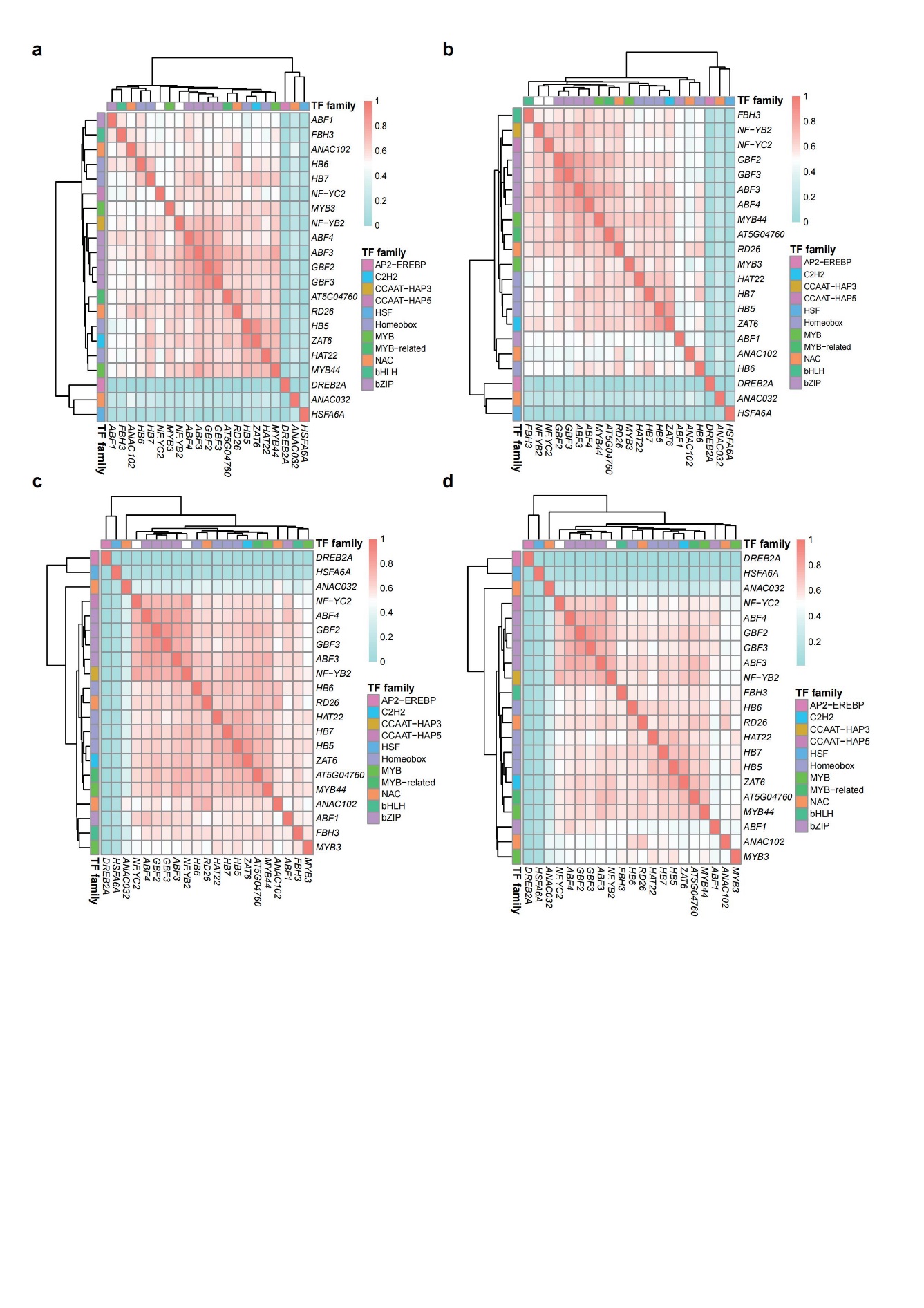
**

**Figure S7. The TMIs and TTIs analysis in the integrated miRNA regulatory network by GOSemSim. a and b** The TMIs in the integrated miRNA regulatory network (**a)** and the total TMIs (**b)**. **c and d** The TTIs in the integrated miRNA regulatory network (**c)** and the total TTIs (**d)**. The different colors around the heatmap represent different TF families.

**
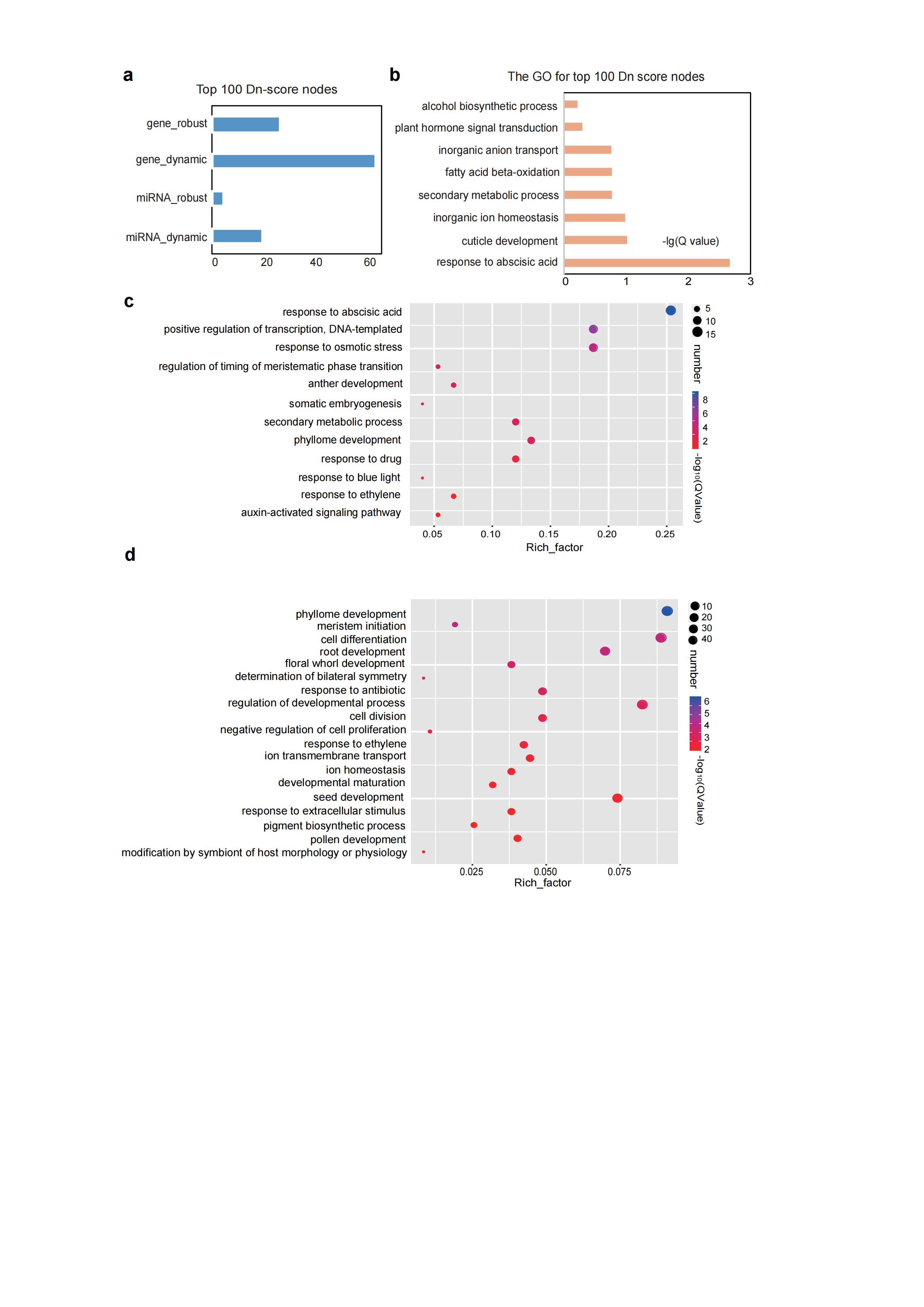
**

**Figure S8.** **The core nodes and GO analysis in the dynamic network. a** Types of the top 100 Dn-score nodes. The distribution of robust genes, dynamic genes, robust miRNAs, and dynamic miRNAs for the top 100 Dn-score nodes in the dynamic network. **b** The GO term of the top 100 Dn-score nodes. **c** GO term analysis of the dynamic nodes in Fig. 2c. **d** GO term analysis of the robust nodes in Fig. 2c.

**
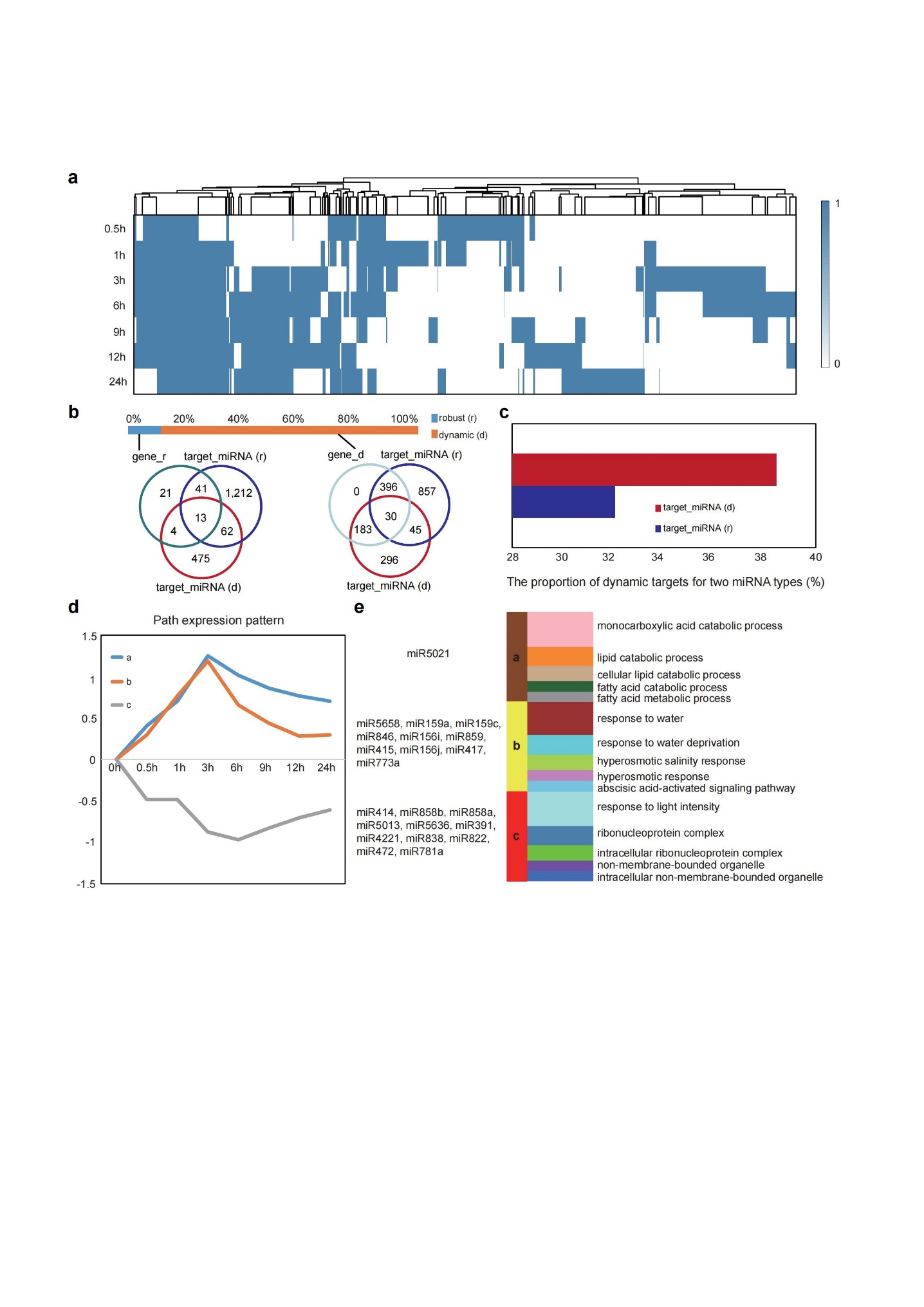
**

**Figure S9. The dynamic edges in the dynamic network. a** Clustering analysis of the dynamic edges shows more variations among the time series data. **b** The distribution of miRNAs and genes in the dynamic network. The upper frame shows the proportion of robust (blue) and dynamic (orange) genes. Venn diagram showing the distribution of dynamic regulatory characteristics of target genes corresponding to different types of miRNAs. ‘d’, ‘dynamic’; ‘r’, ‘robust’. **c** The proportion of dynamic targets regulated by dynamic or robust miRNAs. This value is the dynamic targets divided by the total dynamic (robust) miRNA targets. **d** Line graph depicting the path expression pattern for iDREM analysis. **e** Different expression paths that are regulated by different sets of miRNAs can be separated into different sub-sets. Each sub-set has a specific function.

**
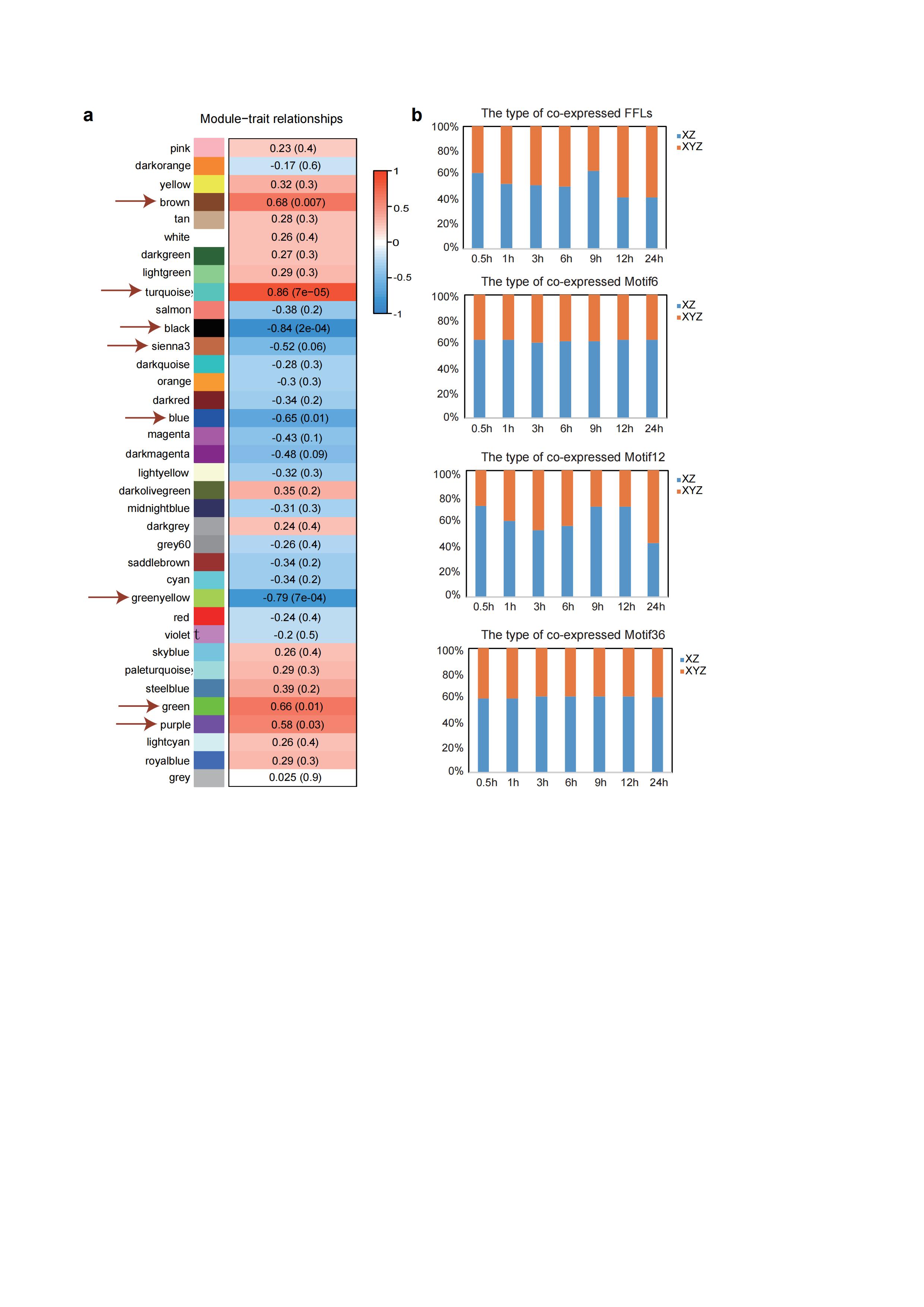
**

**Figure S10. Co-expression analysis of mRNA-miRNAs.** **a** Different color in the middle panel represents different modules. The numbers in the right panel correspond to the correlation coefficient. The arrows represent modules that are significantly correlated with ABA treatment. **b** Co-expression analysis of the nodes in the FFLs, Motif 6, Motif 12, and Motif 36. Orange indicates the co-expressed XYZ and blue indicates the co-expressed XZ.

**
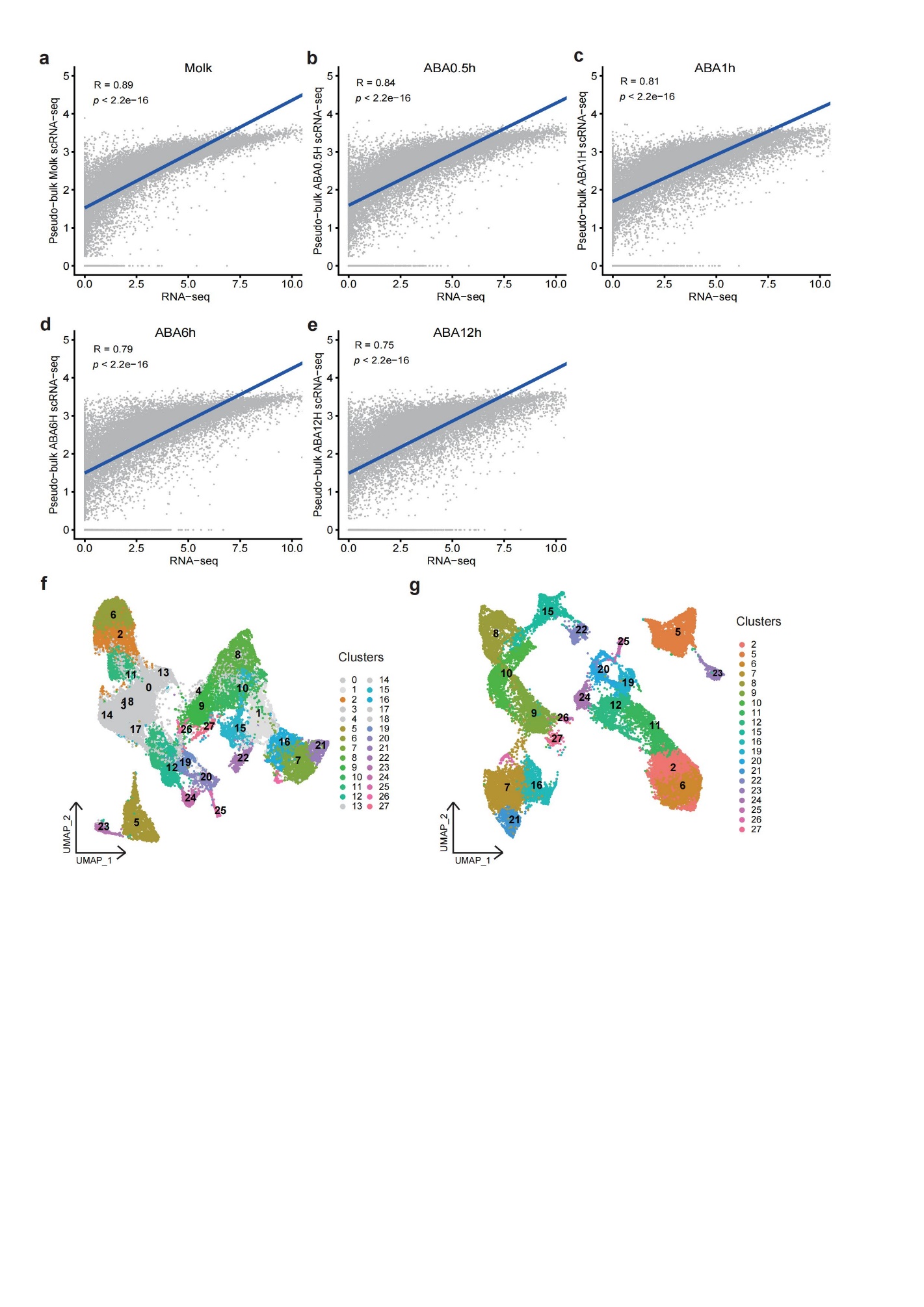
**

**Figure S11. Basic information of scRNA-seq analysis in *Arabidopsis* seedlings**. **a-e**, Correlation of bulk RNA-seq and scRNA-seq expression in each sample. **f** Visualization of 27 clusters with unknown cells (dark grey) and double cells (light grey) using UMAP. Dots, individual cells; n = 50,000 cells; color, clusters. **g** Visualization of the total of 20 clusters after filtering using UMAP. Dots are for individual cells; n = 25,444 cells; colors are for clusters.


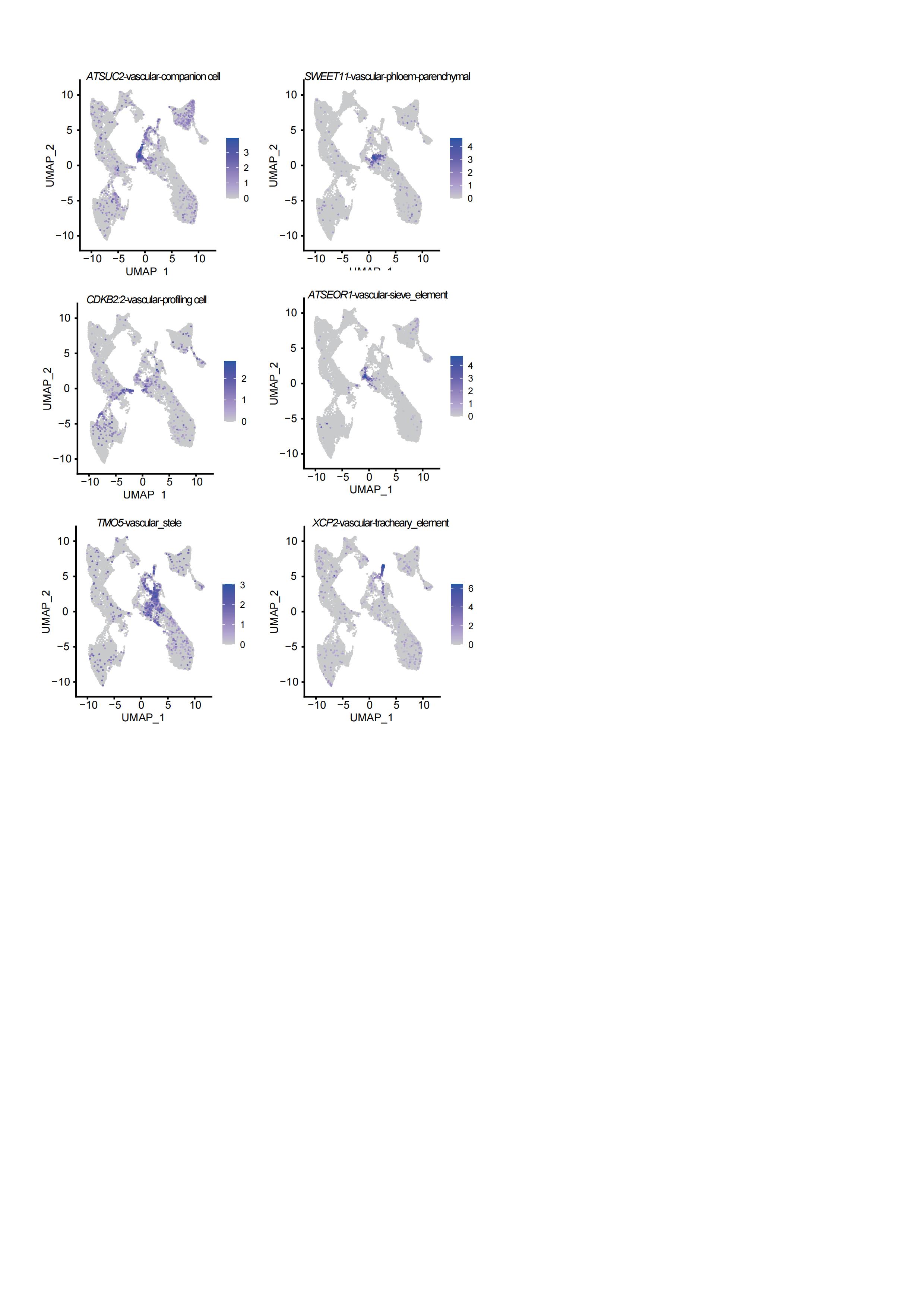


**Figure S12. scRNA-seq analysis of *Arabidopsis* seedlings markers of vascular.** The plots include *ATSU2* in company cell^2^; *SWEET* in phloem parenchymal cell^2^; *CDKB2:2* in profiling cell^2^; *ATSEOR1* in sieve elements^2^; *XCP2* in tracheary elements2; *TMO5* in stele^7^. The full names of selected genes are given in Supplementary Dataset 8.


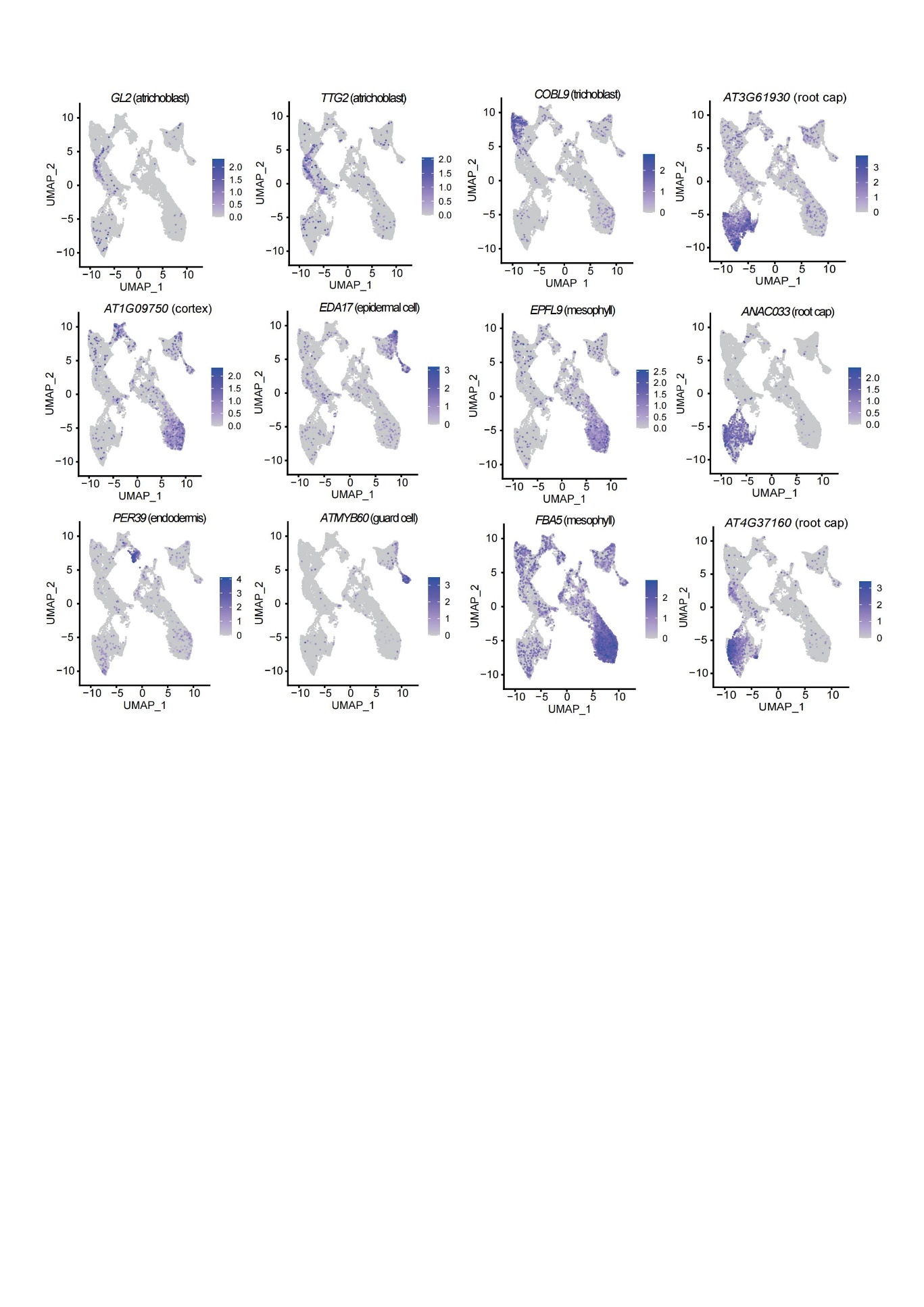


**Figure S13. scRNA-seq analysis of *Arabidopsis* seedlings markers of identified cell types.** The plots including *AT1G09750* in cortex^1^; *PER39* in endodermis^2^; *ATMYB60* in guard cell^3^; *EDA17* in epidermal cell^4^; *GL2* and *TTG* in atrichoblast^1,5^; *COBL9* in trichoblast^6^; *AT3G61930*, *AT4G37160* and *ANAC033* in root cap^7,8^; *EPFL9* and *FBA5* in mesophyll^2^. The full names of selected genes are given in Supplementary Dataset 8.


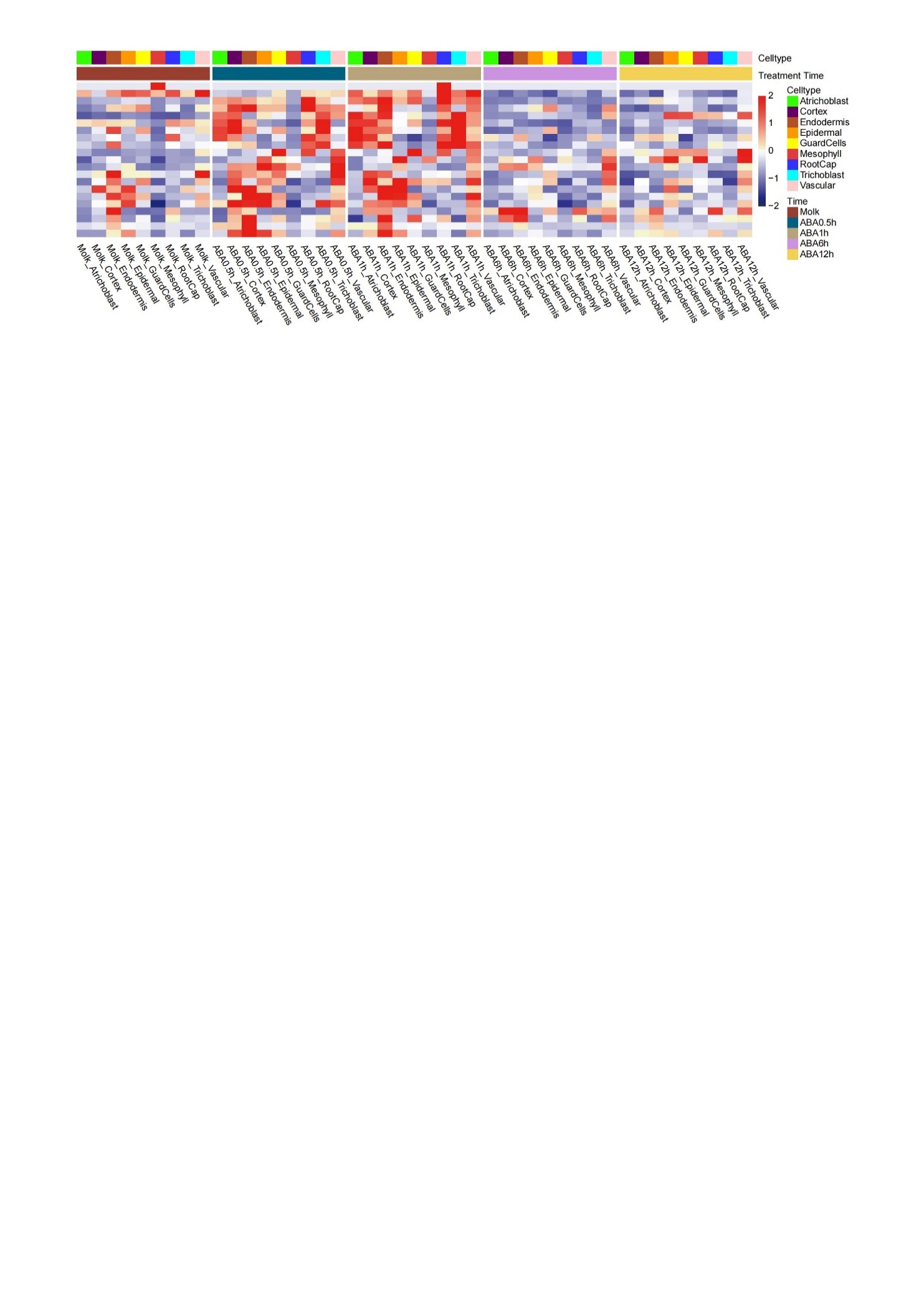


**Figure S14. PPMS analysis of scRNA-seq data in *Arabidopsis* seedlings**. **a** Heatmap shows the expression of 21 TFs in PPMS that are expressed on the different ABA treatments in 9 cell types.


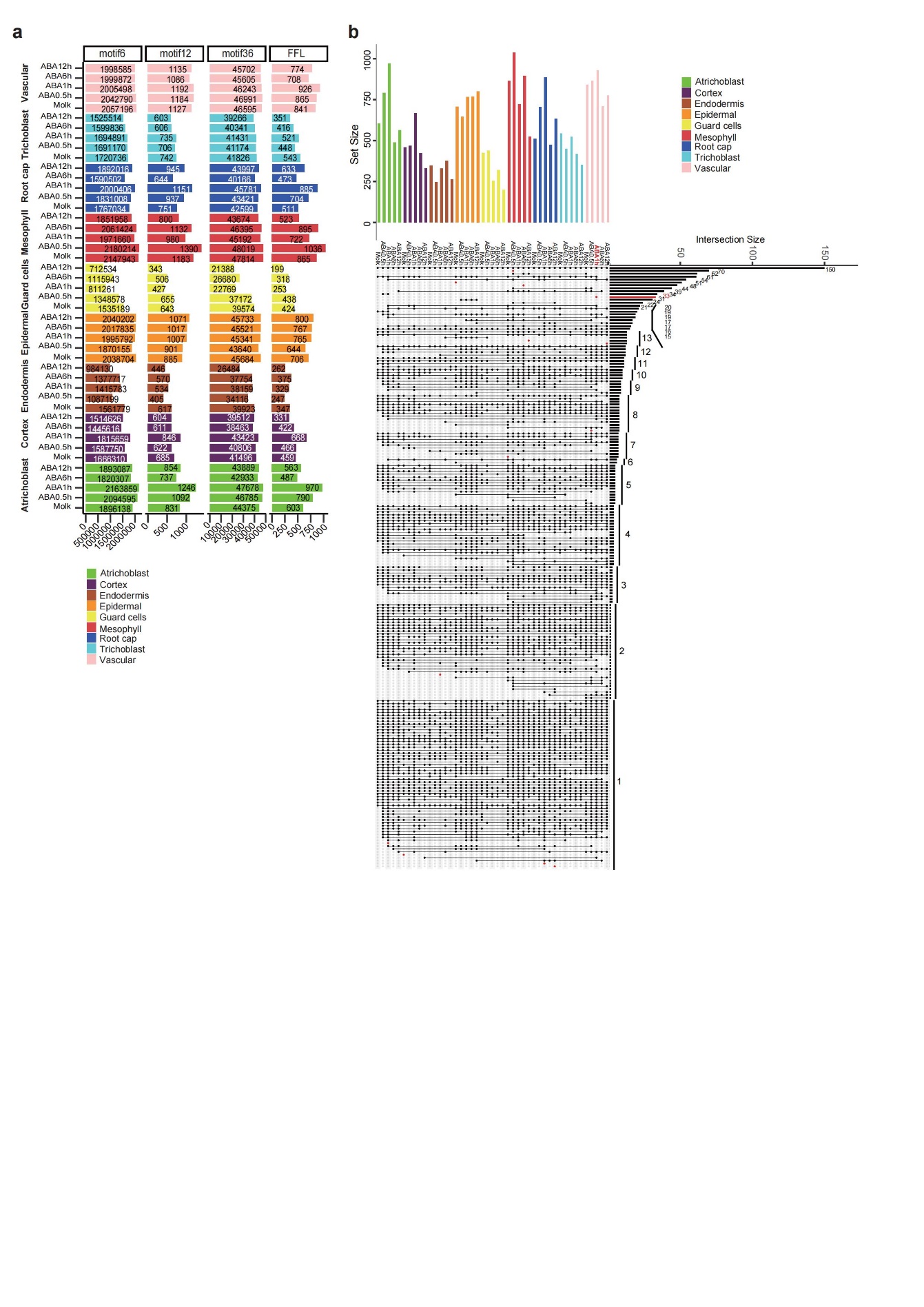


**Figure S15. The statistical analysis of motifs at the single-cell level.**

**a** The formation of four motifs appeared in the 45 groups. The FFL and motif 12 were dynamic in several cell types. **b** Upset analysis of YM-FFLs appearing at 5-time points in 9 cell types. All YM-FFLs were divided into 207 types. There were 33 unique YM-FFLs in vascular of 1h ABA processing.


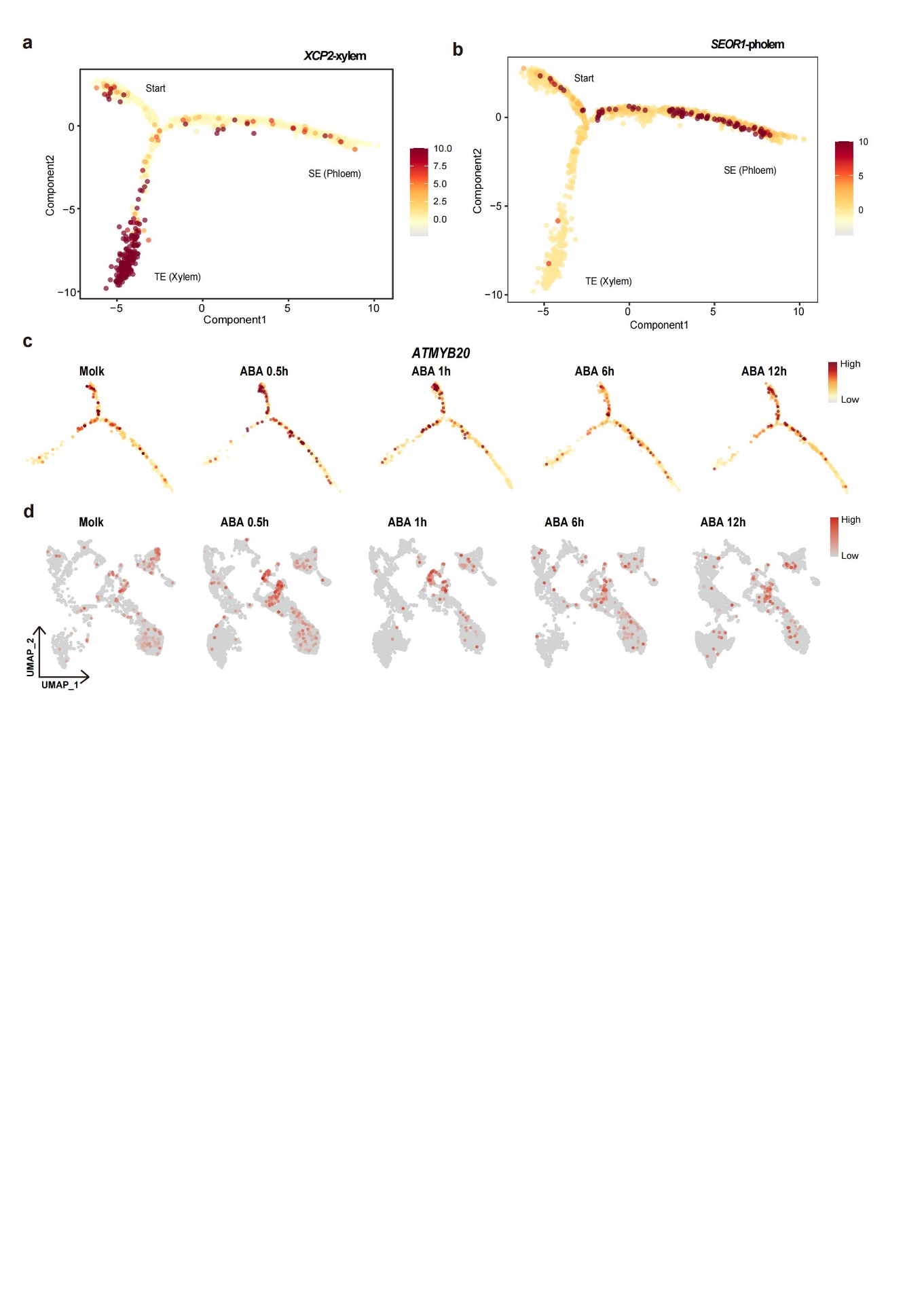


**Figure S16. The monocle2 analysis in vascular. a** The marker in vascular differentiation trajectory of xylem and phloem cells^2^ **b** The marker in vascular differentiation trajectory of phloem cells^2^ **c** *MYB20* expression changes in response to dynamic ABA treatment time points in vascular trajectory. **d** UMAP plot shows the expression pattern of *MYB20* among different time points in all 9 cell types.

**Reference:**

1 Lee, J. Y. *et al.* Transcriptional and posttranscriptional regulation of transcription factor expression in Arabidopsis roots. *Proc Natl Acad Sci U S A* **103**, 6055-6060 (2006).

2 Zhang, T. Q., Chen, Y. & Wang, J. W. A single-cell analysis of the Arabidopsis vegetative shoot apex. *Dev Cell* **56**, 1056-1074 e1058 (2021).

3 Liberman, L. M., Sparks, E. E., Moreno-Risueno, M. A., Petricka, J. J. & Benfey, P. N. MYB36 regulates the transition from proliferation to differentiation in the Arabidopsis root. *Proc Natl Acad Sci U S A* **112**, 12099-12104 (2015).

4 Kurdyukov, S. *et al.* Genetic and biochemical evidence for involvement of HOTHEAD in the biosynthesis of long-chain alpha-,omega-dicarboxylic fatty acids and formation of extracellular matrix. *Planta* **224**, 315-329 (2006).

5 Efroni, I., Ip, P. L., Nawy, T., Mello, A. & Birnbaum, K. D. Quantification of cell identity from single-cell gene expression profiles. *Genome Biol* **16**, 9 (2015).

6 Brady, S. M., Song, S., Dhugga, K. S., Rafalski, J. A. & Benfey, P. N. Combining expression and comparative evolutionary analysis. The COBRA gene family. *Plant Physiol* **143**, 172-187 (2007).

7 Zhang, T. Q., Xu, Z. G., Shang, G. D. & Wang, J. W. A Single-Cell RNA Sequencing Profiles the Developmental Landscape of Arabidopsis Root. *Mol Plant* **12**, 648-660 (2019).

8 Bennett, T. *et al.* SOMBRERO, BEARSKIN1, and BEARSKIN2 regulate root cap maturation in Arabidopsis. *Plant Cell* **22**, 640-654 (2010).
